# Assessment of microbiota in the gut and upper respiratory tract associated with SARS-CoV-2 infection

**DOI:** 10.1186/s40168-022-01447-0

**Published:** 2023-03-03

**Authors:** Jiarui Li, Qiuyu Jing, Jie Li, Mingxi Hua, Lin Di, Chuan Song, Yanyi Huang, Jianbin Wang, Chen Chen, Angela Ruohao Wu

**Affiliations:** 1 Institute of Infectious Diseases, Beijing Ditan Hospital, Capital Medical University and Beijing Key Laboratory of Emerging Infectious Diseases, Beijing, 100015 China; 2 Division of Life Science, The Hong Kong University of Science and Technology, Clear Water Bay, Kowloon, Hong Kong S.A.R. China; 3 School of Life Sciences, Tsinghua University, Beijing, 100084 China; 4 School of Life Sciences, Peking University, Beijing, 100871 China; 5 Biomedical Pioneering Innovation Center (BIOPIC), Peking-Tsinghua Center for Life Sciences, Peking University, Beijing, 100871 China; 6 College of Chemistry and Molecular Engineering, Beijing, 100871 China; 7 Institute for Cell Analysis, Shenzhen Bay Laboratory, Guangdong, 518132 China; 8 Present Address: Biomedical Innovation Center, Beijing Shijitan Hospital, Capital Medical University, 100038 Beijing, China; 9 Department of Chemical and Biological Engineering, The Hong Kong University of Science and Technology, Clear Water Bay, Kowloon, Hong Kong S.A.R. China; 10 Hong Kong Branch of Guangdong Southern Marine Science and Engineering Laboratory (Guangzhou), The Hong Kong University of Science and Technology, Clear Water Bay, Kowloon, Hong Kong S.A.R. China

**Keywords:** SARS-CoV-2, COVID-19, Human microbiota, Upper respiratory tract, Gut

## Abstract

**Background:**

The human microbiome plays an important role in modulating the host metabolism and immune system. Connections and interactions have been found between the microbiome of the gut and oral pharynx in the context of SARS-CoV-2 and other viral infections; hence, to broaden our understanding of host-viral responses in general and to deepen our knowledge of COVID-19, we performed a large-scale, systematic evaluation of the effect of SARS-CoV-2 infection on human microbiota in patients with varying disease severity.

**Results:**

We processed 521 samples from 203 COVID-19 patients with varying disease severity and 94 samples from 31 healthy donors, consisting of 213 pharyngeal swabs, 250 sputa, and 152 fecal samples, and obtained meta-transcriptomes as well as SARS-CoV-2 sequences from each sample. Detailed assessment of these samples revealed altered microbial composition and function in the upper respiratory tract (URT) and gut of COVID-19 patients, and these changes are significantly associated with disease severity. Moreover, URT and gut microbiota show different patterns of alteration, where gut microbiome seems to be more variable and in direct correlation with viral load; and microbial community in the upper respiratory tract renders a high risk of antibiotic resistance. Longitudinally, the microbial composition remains relatively stable during the study period.

**Conclusions:**

Our study has revealed different trends and the relative sensitivity of microbiome in different body sites to SARS-CoV-2 infection. Furthermore, while the use of antibiotics is often essential for the prevention and treatment of secondary infections, our results indicate a need to evaluate potential antibiotic resistance in the management of COVID-19 patients in the ongoing pandemic. Moreover, a longitudinal follow-up to monitor the restoration of the microbiome could enhance our understanding of the long-term effects of COVID-19.

Video Abstract

**Supplementary Information:**

The online version contains supplementary material available at 10.1186/s40168-022-01447-0.

## Background

COVID-19 is an infectious respiratory disease caused by coronavirus SARS-CoV-2. The pandemic has now been ongoing for nearly 2 years since the infection was first reported in Wuhan, China, at the end of 2019. As of December 5, 2021, the ongoing pandemic has affected over 200 countries and regions with more than 266 million confirmed cases, including over 5 million deaths [1]. The emergence of mutations and variants of concern (VOCs) has caused several additional waves of infection and threatens to compromise the effectiveness of existing vaccines and anti-viral drugs [2]. Moreover, SARS-CoV-2 infection can cause long-term effects on human health, with mechanisms largely unknown so far [3].

SARS-CoV-2 binds with angiotensin-converting enzyme 2 (ACE2) on the cell surface, facilitating their entry into the cell and causing infection [4]. Pneumoniae can result when the infection is in alveolar cells, and though the respiratory system is the major target, accumulating evidence shows that SARS-CoV-2 can also infect many other organs. In fact, viral particles and nucleic acids have been detected in diverse specimens, including bronchoalveolar lavage fluid (BALF), sputum, pharyngeal swabs, faeces, blood, and urine [5–7]. Further studies using single-cell RNA sequencing revealed that ACE2 is expressed in a variety of organs and tissues [8–11], and SARS-CoV-2 cell tropism was also identified using postmortem samples in multiple organs [12].

Many studies have demonstrated that unique microbial communities reside on the mucosal surface of the respiratory tract and that these microbiota have complex interactions with the host to maintain balance with the host immune system [13]. Respiratory virus infections can lead to dysbiosis of the microbiota [14, 15] and can predispose patients to secondary bacterial infections, resulting in much higher morbidity and mortality [16, 17]. Aside from local alterations in the respiratory tract, changes in the distal gut microbiota have also been observed during respiratory virus infections, potentially modulated through the so-called “gut-lung axis” [18–20]. Altered respiratory tract and gut microbiota have also been reported during SARS-CoV-2 infections [21–25], and not only were the altered microbiomes associated with disease severity [26–28], but also appeared synchronous between the respiratory tract and the gut [29]. However, these findings were limited by small sample sizes. More detailed comparisons of the microbial composition and function could provide insights into the mechanism of microbial alteration, as well as shed light on the interaction between SARS-CoV-2 infection, microbial community, and host immune system.

We applied a RNA-seq library construction strategy called MINERVA [30], which has greatly reduced hands-on time compared to traditional methods, to capture both the SARS-CoV-2 and metatranscriptomic sequences in samples taken from various body sites of COVID-19 patients. These samples include pharyngeal swabs, sputum, and faeces. In addition to the association of microbiota composition with disease severity in all three sample types, we also observed different patterns of microbial dysbiosis in the upper respiratory tract compared with that of the gut: the gut microbiota composition is highly heterogeneous among patients and its alteration seems directly associated with SARS-CoV-2 viral abundance. In addition, microbial functions between the URT and gut are also distinct, with a high abundance of stress and toxin-related gene expression found in the URT, compared to the loss of carbohydrate metabolism and short-chain fatty acids (SCFA)-generating genes in the gut microbiota of COVID-19 patients. These results suggest that the URT microbiota may render a high risk of antibiotic resistance; while the gut microbiome could be more sensitive to SARS-CoV-2 abundance and become more unstable. As such, we posit that in the care of COVID-19 patients, SARS-CoV-2-associated antimicrobial resistance, control of secondary infection, and supportive maintenance of microbial homeostasis warrant more clinical attention.

## Methods

### Patients and clinical samples

**Fig. 1:**
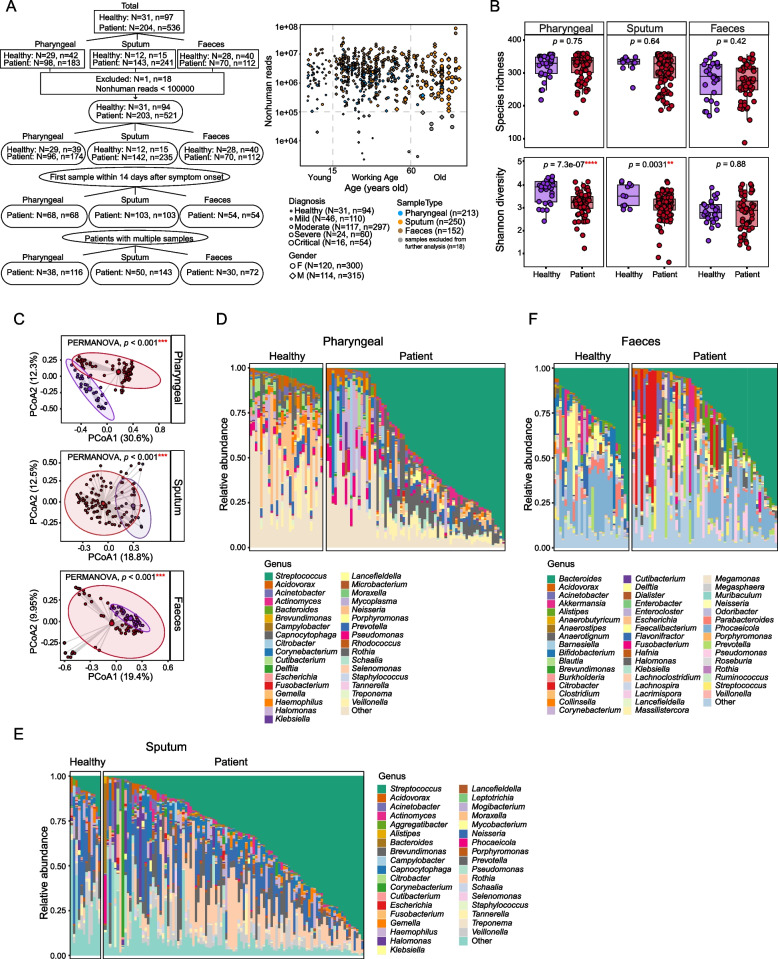
Overview of the dataset and microbial composition in different sample types of COVID-19 patients. **A** Summary of sample and patient information used in this study (left) and distribution of sequencing depth for each sample (right). Uppercase N indicates the number of patients and lower-case n represents the number of samples. Besides disease diagnosis, gender, and sample types, samples were also divided into young (<15 years old), working age (15–60 years old), and old (≥60 years old) groups based on patient age. Samples with nonhuman reads less than 100,000 were labeled in gray and excluded from further analysis. **B** Comparison of alpha diversity between healthy controls and COVID-19 patients. Wilcox rank-sum test was used. **C** PCoA and PERMANOVA analysis of the microbial composition using Bray-Curtis distance in healthy controls and COVID-19 patients. Detailed microbial community was also profiled at genus level for pharyngeal (**D**), sputum (**E**), and faeces (**F**) samples. Only the first sample of each subject was used for comparison in **B**–**F**

From January 23, 2020, to April 20, 2020, 204 patients were enrolled in this study according to the 7th guideline for the diagnosis and treatment of COVID-19 from the National Health Commission of the People’s Republic of China [31]. All patients, diagnosed with COVID-19, were hospitalized in Beijing Ditan Hospital and classified into three severity degrees: mild, moderate, and severe illness, according to the same aforementioned guidelines [31]. Briefly, mild cases are those with mild clinical symptoms, and there was no sign of pneumonia on imaging. Moderate cases are those showing fever and respiratory symptoms with radiological findings of pneumonia. Severe cases include adult cases meeting any of the following criteria: (1) respiratory distress (≥30 breaths/min), (2) oxygen saturation ≤93% at rest, (3) arterial partial pressure of oxygen (PaO_2_)/fraction of inspired oxygen (FiO_2_) ≤300 mmHg, (4) respiratory failure and requiring mechanical ventilation, (5) shock, and (6) with other organ failures that require ICU care. In total, we collected 536 samples, including 183 pharyngeal swabs, 241 sputa, and 112 fecal samples from these patients. We have also collected 97 samples from 31 healthy donors, including 42 pharyngeal swabs, 15 sputa, and 40 faeces (Fig. 1A).

This study was approved by the Ethics Committee of Beijing Ditan Hospital, Capital Medical University (No. KT2020-006-01), and consent was obtained from all participating patients in accordance with the approved ethics protocol.

### RNA extraction, library construction, and sequencing

For all the clinical samples, nucleic acid extraction was performed in a BSL-3 laboratory. Samples were deactivated by heating at 56°C for 30 min before extraction. Total RNA was extracted using QIAamp Viral RNA Mini Kit (Qiagen) following the manufacturer’s instructions. After nucleic acid extraction, ribosomal RNA (rRNA) was removed by ribosomal DNA (rDNA) probe hybridization and RNase H digestion, followed by DNA removal through DNase I digestion, using MGIEasy rRNA removal kit (BGI, Shenzhen, China). The final elution volume was 12–20 μl for each sample. The sequencing library was constructed following the MINERVA protocol [30]. Briefly, 2.7 μl RNA from rRNA and DNA removal reaction was used for standard SHERRY reverse transcription [32], with the following modifications: (1) 10 pmol random decamer (N10) was added to improve coverage, and (2) initial concentrations of dNTPs and oligo-dT (T30VN) were increased to 25 mM and 100 μM, respectively. The RNA/DNA hybrid was tagmented in TD reaction buffer (10 mM Tris-Cl pH 7.6, 5 mM MgCl_2_, 10% DMF) supplemented with 3.4% PEG8000 (VWR Life Science, Cat.No.97061), 1 mM ATP (NEB,Cat.No. P0756), and 1U/μl RNase inhibitor (TaKaRa, Cat.No. 2313B). The reaction was incubated at 55°C for 30 min. A 20-μl tagmentation product was mixed with 20.4 μl Q5 High-Fidelity 2X Master Mix (NEB, Cat.No. M0492L), 0.4 μl SuperScript II reverse transcriptase, and incubated at 42°C for 15 min to fill the gaps, followed by 70°C for 15 min to inactivate SuperScript II reverse transcriptase. Then, indexing PCR was performed by adding 4 μl 10μM unique dual index primers and 4 μl Q5 High-Fidelity 2X Master Mix, with the following thermo profile: 98°C 30 s, 18 cycles of [98°C 20 s, 60°C 20 s, 72°C 2 min], 72°C 5 min. The PCR product was then purified with 0.8x VAHTS DNA Clean Beads (Vazyme, Cat. No. N411). These libraries were sequenced on Illumina NextSeq 500 with 2x75 paired-end mode for metagenomic analysis.

### Raw data processing and microbial taxonomy assignment

For the raw sequencing reads, bases with a quality lower than 20 were firstly trimmed in a k-mer-based strategy using BBmap (version 38.68) [33]. Reads with a length shorter than 20bp were discarded. Qualified reads were then mapped to the human genome reference (GRCh38) using STAR (version 2.6.1d) [34] with default parameters. All unmapped reads were collected using samtools (version 1.3) [35], and then, rRNA reads were removed using SortMeRNA (version 2.1b) [36] based on SILVA and Rfam databases. Samples with nonhuman reads less than 100k after rRNA removal were excluded from the following analysis to ensure enough sequencing depth for microbiota profiling. The microbial taxonomy assignment was done by Kraken2 [37]. Custom reference was built from all complete bacterial, viral, and any assembled fungal genomes downloaded from the NCBI RefSeq database (viral and fungal genomes were downloaded on February 4, 2020, and bacterial genomes were downloaded on November 14, 2018). There were 11,174 bacterial, 8997 viral, and 308 fungal genomes, respectively. Taxa with only 1 mapped read were excluded to avoid random false positives. Decontamination was performed first by PERFect using the “permutation filtering” method [38]; then, the left microbes for each sample were further filtered based on non-template controls as described by Shen *et al*. with modifications [39]. Briefly, only microbes satisfying the following criteria would be considered true signals and would be kept for the following analysis: (1) at least 4-fold of that in non-template controls and (2) with relative abundance ≥1%.

### Analysis of *Halomonas* species

Nonhuman reads from samples with high abundance (≥3% of total bacterial reads) of *Halomonas* detected were mapped against genomic references of all *Halomonas* species downloaded from the NCBI RefSeq database using Bowtie2 [40]. We extracted mapped reads for co-assembly using Megahit [41] and metaSpades [42] at the same time. In total, we got 4182 contigs with a total length of 2.67Mbp and the N50 value as 896bp from Megahit; metaSpades gave relatively better results, with 6577 contigs, a total length of 2.67Mbp and N50 as 1295bp. To identify species, we aligned contigs against all *Halomonas* species genomes using Megablast (-evalue 1e−10; -qcov_hsp_perc 70; -perc_identity 90) [43]. The genomic similarity between assembled contigs with top species from blast results was compared using an in-silicon DNA-DNA hybridization strategy [44]. Comparative circular genomes were visualized using CGView [45].

### Microbial function analysis

Reads after the removal of human and rRNAs were used for microbial function analysis using HUMAnN2 (version 2.8.1) [46]. Gene families were first identified based on UniRef90 database and then regrouped to KEGG Orthogroups (KO) and normalized as relative abundance. Gene families with relative abundance >1e−5 in more than 30% of the samples were used for the comparison between different disease groups using linear discriminant analysis by LEfSe (version 1.0.7) [47]. Finally, differential features with relative abundance >5e−5 in more than 30% of samples of each group and FDR-adjusted *p* values <0.05, as well as log-transformed LDA≥2 were kept. The functional category was annotated based on UniProt database [48].

### Quantification and statistical analysis

Permutational multivariate analysis of variance (PERMANOVA) was applied to assess meta-factors associated with microbial composition in different sample types using R vegan package. Differential genera and species in each group were identified by linear discriminant analysis using LEfSe, only microbes with FDR-adjusted *p* values <0.05 were denoted as significant. Kruskal-Wallis test was applied for multi-group comparisons, and Wilcox rank sum test was used as post hoc test between two groups if not specifically stated. The Spearman’s correlation coefficients were transformed using Fisher’s *Z* transformation for comparison. All statistical analysis and visualization were performed in R (version 3.5.1).

## Results

### Overview of the dataset

In total, we collected 536 samples from 204 COVID-19 patients and 97 samples from 31 healthy donors, of which 225 samples were pharyngeal swabs, 256 samples were sputum, and 152 samples were faeces (Fig. 1A, left). To ensure adequate sequencing depth for profiling microbial composition, samples with fewer than 100,000 reads after the removal of low-quality reads, human reads, and rRNA reads were excluded (Fig. 1A, right). Finally, 521 samples from 203 patients and 94 samples from 31 healthy donors were kept for the following analysis. The summary characteristics of the subjects are shown in Table 1. To avoid potential bias introduced by multiple sampling from the same patients at different time points, we selected the first sample of each patient that was collected within 14 days since symptom onset as a representative to profile the microbial composition and function. Using patients with multiple samples available, we further checked the dynamics of microbial signatures. All patient samples were collected before their discharge from the hospital. Detailed information about each sample is listed in Supplementary Table S1. We also included non-template controls (NTCs) during nucleic acid extraction, library construction, and sequencing to profile potential environmental or reagent contaminations. Microbes detected in non-template controls are shown in Supplementary Table S2.

**Table 1:** Summary characteristics of the study cohort

**Patients with pharyngeal swab samples**				
**Clinical indexes**	**Healthy controls (*N*=29,** ***n*=39)**	**Patient (N=96, n=174)**		
		**Mild (*N*=19,** ***n*=31)**	**Moderate (*N*=51,** ***n*=73)**	**Severe (*N*=26,** ***n*=70)**
**Age**	**33 (23–42)**	**22 (8–28)**	**37 (23–41)**	**71 (59–79)**
**Sex (female/male)**	**18/11**	**9/10**	**27/24**	**10/16**
**Antibiotic usage**	**0**	**2**	**20**	**13**
**Anti-viral drug usage**	**0**	**13**	**78**	**22**
**Patients with sputum samples**				
**Clinical indexes**	**Healthy controls (*N*=12,** ***n*=15)**	**Patients (*N*=142,** ***n*=235)**		
		**Mild (*N*=27,** ***n*=39)**	**Moderate (*N*=89,** ***n*=156)**	**Severe (*N*=26,** ***n*=40)**
**Age**	**32 (25–36)**	**23 (18–28)**	**38 (24–48)**	**62 (47–73)**
**Sex (female/male)**	**7/5**	**15/12**	**46/43**	**10/16**
**Antibiotic usage**	**0**	**4**	**20**	**13**
**Anti-viral drug usage**	**0**	**18**	**78**	**22**
**Patients with stool samples**				
**Clinical indexes**	**Healthy controls (*N*=28,** ***n*=40)**	**Patient (*N*=70,** ***n*=112)**		
		**Mild (*N*=22,** ***n*=40)**	**Moderate (*N*=44,** ***n*=68)**	**Severe (*N*=4,** ***n*=4)**
**Age**	**32 (7–40)**	**22 (6–26)**	**35 (25–46)**	**52 (36–69)**
**Sex (female/male)**	**15/13**	**12/10**	**19/25**	**1/3**
**Antibiotic usage**	**0**	**3**	**8**	**4**
**Anti-viral drug usage**	**0**	**17**	**38**	**3**

### Altered microbiota in different sample types from COVID-19 patients

Multiple studies have reported the alteration of respiratory tract microbiome in COVID-19 patients, in terms of both alpha and beta diversity [24, 49]. In our dataset, there is no difference in species richness (number of species) in all three sample types between patients and healthy controls, but Shannon diversity was significantly reduced in respiratory tract samples, including both pharyngeal swabs and sputum of COVID-19 patients (Fig. 1B). We also did not observe any difference in the alpha diversity of fecal samples, which somewhat differs from the results reported by others that gut microbiome of COVID-19 patients had significantly reduced alpha diversity [22, 25]. However, when we then compared the beta diversity between patients and healthy controls, patient samples form a distinct group from controls on principle coordinate analysis (PCoA) using Bray-Curtis distance (PERMANOVA test, *p* < 0.001) for pharyngeal, sputum, and fecal samples (Fig. 1C). This suggests that even though there is no difference in terms of the alpha diversity, COVID-19 patients’ gut microbiomes were indeed altered compared to controls. We further profiled the microbial composition at the genus level in healthy donors and COVID-19 patients for all three sample types. In pharyngeal and sputum samples, there is a striking increase of *Streptococcus*, the most abundant genus in the patient group (Fig. 1D, E). In fecal samples, *Bacteroides* is the most abundant genus but shows no obvious difference between patients and healthy controls; rather, the overall microbial composition of patient samples shows distinctive patterns compared to healthy controls, as exemplified by a decrease in *Fecalibacterium* and expansion of *Acinetobacter, Citrobacter,* and *Pseudomonas* (Fig. 1F).

### Alteration of microbial composition in COVID-19 patients is associated with disease severity

**Fig. 2:**
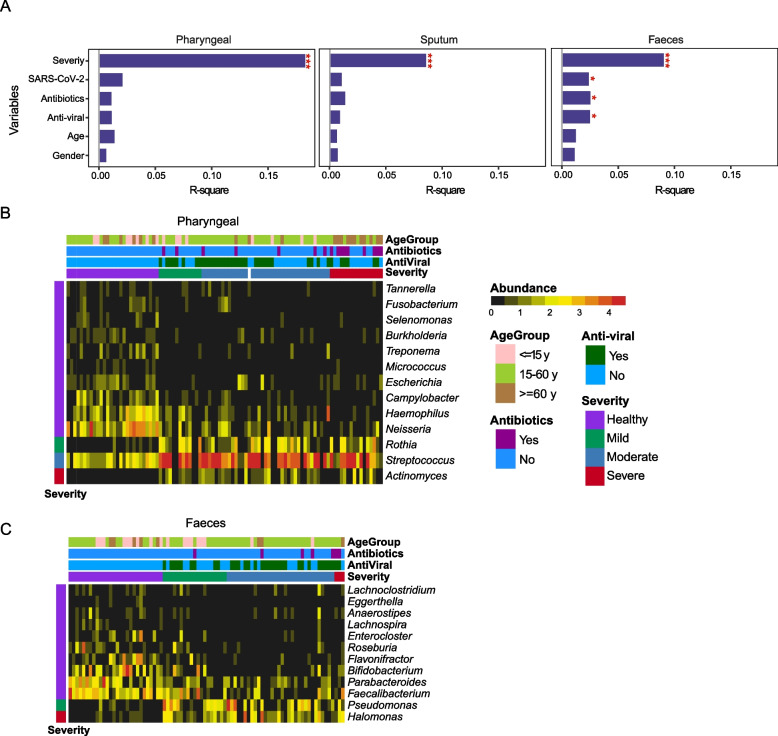
Altered microbial composition associated with disease severity. **A** PERMANOVA test of meta-factors potentially associated with microbial composition in all three sample types. Representative genera associated with disease severity were identified by LEfSe in pharyngeal (**B**) and faeces (**C**) samples, respectively

Since there are microbial composition changes in both the respiratory tract and gut samples of COVID-19 patients, we further assessed the effect of meta-factors associated with these alterations and found that the microbial composition is significantly affected by disease severity in all three sample types (PERMANOVA; Fig. 2A). Consistently, Shannon diversity was significantly reduced in pharyngeal and sputum samples of COVID-19 patients, irrespective of the disease severity (Supplementary figure S1A). The situation was more complex in fecal samples, while there is no reduction in the alpha diversity, there are significant effects on the microbial composition attributed to SARS-CoV-2 abundance as well as antibiotic and anti-viral treatments (Fig. 2A and Supplementary figure S1A).

To further identify differential microbes associated with disease severity, we performed linear discriminant analysis using LEfSe in pharyngeal samples and found *Tannerella*, *Fusobacterium*, *Selenomonas*, *Burkholderia*, *Treponema*, *Micrococcus*, *Escherichia*, *Campylobacter*, *Haemophilus*, and *Neisseria* to be depleted in COVID-19 patients; *Rothia*, *Streptococcus*, and *Actinomyces* were enriched (Fig. 2B). We also identified differentially represented bacterium in sputum samples: *Treponema*, *Delftia*, *Porphyromonas*, *Tannerella*, *Haemophilus*, and *Neisseria* showed decreased abundance in patients, especially in those with severe symptoms, while *Capnocytophaga* and *Streptococcus* were elevated (Supplementary figure S1B). Although the origin of the microbes found in sputum samples cannot be precisely pinpointed as upper or lower respiratory tract, we found that the same bacterial species were altered in both pharyngeal (Supplementary figure S1C) and sputum (supplementary figure S1D) samples of severe symptom patients. Multiple *Streptococcus* species were enriched in patient samples, and some of them are reported to be normal flora colonizing the oral cavity or respiratory tract, but are also capable of causing opportunistic infections [13, 50, 51]. In fecal samples, *Lachnoclostridium*, *Eggerthella*, *Anaerostipes*, *Lachnospira*, *Enterocloster*, *Roseburia*, *Flavonifractor*, *Bifidobacterium*, *Parabacteroides*, and *Faecalibacterium* were reduced in patients, whereas *Pseudomonas* and *Halomonas* were enriched (Fig. 2C). At species level (Supplementary figure S1E), multiple depleted microbes were reported to be involved in the generation of SCFA and play important role in modulating gut health and response to inflammation, such as *Parabacteroides distasonis*, *Roseburia hominis*, *Faecalibacterium prausnitzii*, and many *Bifidobacterium* species [52, 53].

For microbes found to be associated with disease severity (Fig. 2B, C, and Supplementary figure S2B), increased abundance in the corresponding severity group was observed even though not statistically significant (Supplementary figure S1F). We noticed that there are species enriched in COVID-19 which are not commonly detected in human faeces, including the *Pseudomonas fluorescens* group and unclassified *Halomonas* (Supplementary figure S1E). *Pseudomonas fluorescens* generally has low virulence but can also cause human infection infrequently [54]. *Halomonas* species are usually found in marine or saline environments, as they thrive under high salinity environments. However, some strains were reported to cause nosocomial infections and contaminations in hospital settings [55]. To more accurately identify the *Halomonas* species in our samples, we separately mapped non-human reads from samples with a high abundance of *Halomonas* (≥3% of total bacterial reads) to all *Halomonas* genomes downloaded from NCBI RefSeq database and performed meta-assembly with two different approaches. To achieve species-level identification, we then blasted the assembled contigs against the known *Halomonas* genomes. Most of the hits had ~99% identity, confirming the existence of *Halomonas* in our samples (Supplementary figure S2A). We further compared the genomic similarity between assemblies and top species identified from blast results (Supplementary figure S2B). The similarity between assemblies generated using different methods was 97.9%, indicating comparable results from both. There were no species with more than 70% similarity identified. Interestingly, several species reported to be associated with infection in clinical environments were also detected in our samples, including *H. stevensii*, *H. johnsoniae*, and *H. hamiltonii* [55, 56]. Genomes with more than 60% similarity were visualized in Supplementary figure S2C.

### Gut microbiota alterations are associated with SARS-CoV-2 abundance

**Fig. 3:**
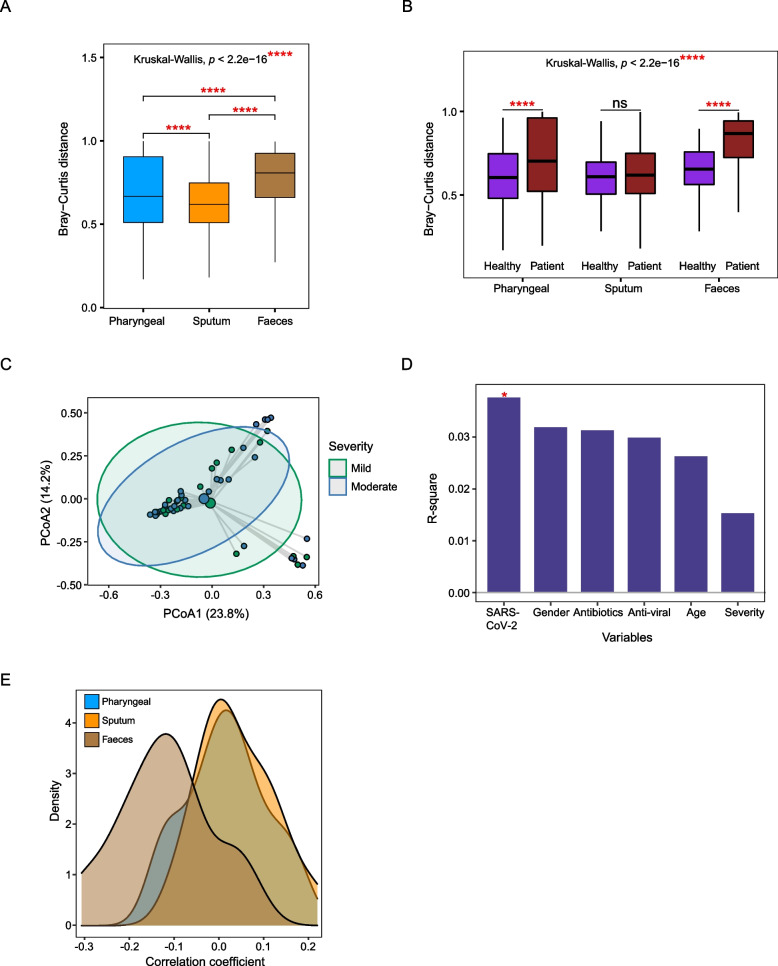
Comparison of the dysbiosis patterns of microbial composition in respiratory tract and gut samples. **A** Comparison of Bray-Curtis distance between patients and healthy controls in three sample types. **B** Comparison of the within-patient and within-healthy control Bray-Curtis distance in three sample types. **C** PCoA analysis of the microbial composition in faeces samples from mild and moderate patients. **D** PERMANOVA test to identify meta-factors potentially associated with the microbial composition in faeces samples from mild and moderate patients. **E** Distribution of the correlation coefficients between SARS-CoV-2 and representative species in three sample types. Kruskal-Wallis test was used for multiple-group comparison and Wilcox rank-sum test was used for post hoc two-group comparison

We have observed different alteration patterns of gut microbiota in COVID-19 patients from that of the respiratory tract when comparing alpha and beta diversity. Meanwhile, patient gut microbiomes were affected by multiple factors, including disease severity, SARS-CoV-2 abundance, as well as antibiotic and anti-viral treatment (Fig. 2A). Moreover, there is a greater Bray-Curtis distance between patients and corresponding healthy controls in fecal samples (Fig 3A), indicating that the gut microbiota may be more severely disrupted by SARS-CoV-2 infection. We also calculated Bray-Curtis distances for samples within the patient and healthy control groups, respectively. Generally, the within-patient Bray-Curtis distance is higher than the within-control distance for all three sample types; moreover, the degree of increase is larger in fecal samples (Fig. 3B), which suggested that gut microbial composition of COVID-19 patients is more dispersed than the respiratory tract microbiota.

To further explore potential mechanisms associated with the different alteration patterns between URT and gut microbiota, we assessed the effects of meta factors, including disease severity, age, gender, SARS-CoV-2 abundance, antibiotic, and anti-viral treatment using patient samples. Only patients with mild or moderate symptoms were included since there are insufficient numbers of fecal samples from severe patients. Our results show that in pharyngeal and sputum samples, there is no significant difference in the microbial composition between mild and moderate patients (Supplementary figure S3A and S3C); at the same time, no significant associations were found among the tested factors (Supplementary figure S3B and S3D). In contrast, microbial composition in fecal samples was significantly associated with SARS-CoV-2 abundance (Fig. 3C and D), which suggests that gut microbiota might be more vulnerable to disruption by SARS-CoV-2 infection. We then checked for the correlation between the abundance of SARS-CoV-2 and that of the major species from differential analysis (Supplementary figure S1C, S1D, and S1E) and found a stronger negative correlation in fecal samples (Fig. 3E). This result was also confirmed at the genus level (Supplementary figure S3E). Together, these findings suggest that alteration of gut microbiota was more likely to be directly associated with SARS-CoV-2 abundance. In addition to disease progression, gut microbiota could be more easily affected by therapies, showing greater dispersion than upper respiratory tract microbiome in COVID-19 patients.

### Microbial composition remains relatively stable during the study period

We checked the dynamics of the microbial composition for patients with multiple samples available. All samples were collected during the patients’ hospitalization period. The microbial composition profiled by representative genera from differential analysis remained relatively stable in samples from the same patient, irrespective of disease severity, and sample type (Supplementary figure S4A, S4B, and S4C). We also checked the dynamics of the average Bray-Curtis distance to healthy controls for each patient. There were fluctuations in a subset of patients, such as P17, P37, P60, P102, and P103, in terms of pharyngeal swabs (Supplementary figure S5A); P17, P137, P161, P164, P165, P168, P169, and P198 in terms of sputum (Supplementary figure S5B) as well as P137, P145, and P239 in terms of fecal samples (Supplementary figure S5C). However, generally, the Bray-Curtis distance remained relatively stable during the sampling period.

### Altered microbial function in COVID-19 patients

In addition to composition, we also assessed the function of microbial community potentially associated with disease severity using the LEfSe method. Generally speaking, different patterns of functional dysbiosis were observed in the respiratory tract and gut microbiota of COVID-19 patients. In the respiratory tract, the microbial community conferred a high abundance of stress-response and toxin genes, while gut microbiota was mainly found with loss of carbohydrate metabolism and SCFA-generating pathways and also with enrichment of stress response-related genes.

**Fig. 4:**
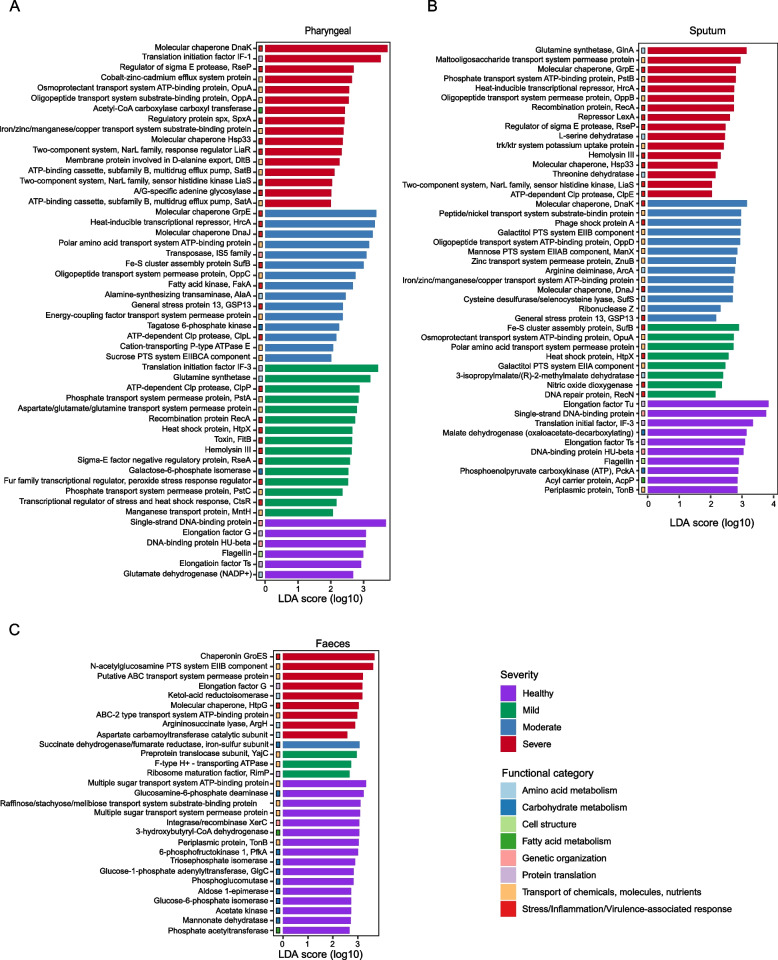
Altered microbial function in COVID-19 patients. Differentially enriched microbial functions associated with disease severity were identified by LEfSe in pharyngeal (**A**), sputum (**B**), and faeces (**C**) samples

Specific to pharyngeal samples, multiple genes related to fundamental cellular activities were enriched in healthy controls, such as glutamate dehydrogenase (NADP+), which is involved in amino acid metabolism; and factors related to protein translation, such as elongation factor G and Ts; as well as cellular components flagellin (Fig. 4A). In contrast, multiple genes related to the transportation of different molecules were found to be enriched in patient samples, such as manganese transport protein, phosphate transport system permease, sucrose PTS system EIIBCA component, iron/zinc/manganese/copper transport system substrate-binding protein, oligopeptide transport system permease protein, and osmoproctectant transport system ATP-binding protein. Moreover, multidrug efflux pump SatA and SatB, components of norfloxacin and ciprofloxacin ABC transport [57, 58], were found enriched in severe patients, indicating a higher risk of antibiotic resistance in the microbial community profiled from patient samples. Genes related to bacterial response to stress/inflammation and virulence-related genes were also found to be abundant in patient samples regardless of the disease severity (Fig. 4A). Similar in sputum samples, functions related to normal cellular activities, including carbohydrate and fatty acid metabolism, protein translation, and genetic organization, were enriched in healthy controls. In patient samples, multiple genes related to the transportation of molecules, such as metal ions, oligopeptides, and amino acids, as well as genes related to stress response and virulence were found highly abundant (Fig. 4B).

Many of the upregulated genes found in the respiratory tract microbiota of COVID-19 patients are associated with bacterial response to diverse stresses. RseA is a negative regulator of the sigma E factor, whose function is central to the response to envelope stress [59, 60]. General stress protein 13 was found to be in association with the 30S subunit of the ribosome and can be induced by heat shock, salt stress, oxidative stress, glucose, and oxygen limitations [61]. Clp protease plays a central role in proteolysis and is involved in bacterial adaptation to various environmental stresses [62]. Fatty acid kinase FakA is involved in lipid metabolism and is important for the activation of the SaeRS two-component system and secreted virulence factors like α-hemolysin [63]. SufB is a component of the Suf system, which is a specialized pathway for Fe-S cluster assembly under iron starvation or oxidative stress [64]. DNA glycosylase MutY mainly functions to correct DNA G-A mispairs from oxidative damage [65]. LiaR is a component of the LiaFSR system, which is a gene regulatory system important for response to cell membrane stress in Gram-positive bacteria [66]. Toxin FitB is a component of the type-II toxin-antitoxin system and plays a role in the speed with which bacteria traverse human epithelial cells [67]. Hemolysin-III is a potent pore-forming toxin [68]. Taken together, the enrichment of these genes in patient samples suggested that the microbial community was underlying stressful situations, which might be caused by SARS-CoV-2 infection or other factors, such as treatment or host inflammation.

In fecal samples, healthy controls were observed with enriched fatty acid metabolism genes, such as phosphate acetyltransferase and 3-hydroxybutyryl-CoA dehydrogenase; and carbohydrate metabolism genes, such as mannose dehydratase, acetate kinase, glucose-6-phosphate isomerase, aldose 1-epimerase, phosphoglucomutase, gluose-1-phosphate adenylyltransferase, triosephosphate isomerase, 6-phosphofructokinase 1 and gluocosamine-6-phosphate deaminase; and carbohydrate transportation proteins, such as raffinose/stachyose/melibiose transport system substrate-binding protein and components of multiple sugar transport system. Some of the genes are known to be involved in the pathways generating short-chain fatty acids. Phosphate acetyltransferase catalyzes the reversible interconversion of acetyl-CoA and acetyl phosphate, which is related to acetate synthesis [69]. Acetate kinase can catalyze the formation of acetyl phosphate from acetate and ATP and also the reverse reaction to favor the formation of acetate. A 3-hydroxybutyryl-CoA dehydrogenase converts 3-hydroxybutanoyl-CoA to acetoacetyl-CoA and is involved in the butanoate metabolism [70, 71]. These functions were depleted in patient fecal samples. In patients, the elevated genes were mainly related to amino acid metabolisms, such as aspartate carbamoyltransferase catalytic subunit, argininosuccinate lyase, and ketol-acid reductoisomerase and other molecule transporting proteins, such as F-type H+ transporting ATPase, preprotein translocase subunit YajC, ABC transport system, and *N*-acetylgucosamine PTS system EIIB components. At the same time, stress response-related genes were also found to be enriched in patient fecal samples, such as molecular chaperone HtpG and chaperonin GroES (Fig. 4C).

## Discussion

We systematically evaluated the microbiota in diverse sample types of COVID-19 patients. There are alterations directly associated with disease severity in the URT, represented mainly by pharyngeal swab samples, and also in the gut microbiome. Moreover, the URT and gut microbiota show different patterns of alterations. There is reduced microbial diversity in both pharyngeal swabs and sputum samples, which may be due to the loss of normal flora and expansion of *Streptococcus*. This echoes a previous study that discovered *Streptococcus* to be dominant in the URT of recovered COVID-19 patients and *S. parasanguinis* to be correlated with prognosis in non-severe subjects [28]. In gut samples, we saw a depletion of beneficial microbes, including *Roseburia*, *Bifidobacterium*, *Parabacteroides*, and *Faecalibacterium* in patients, which are well-known SCFA-generating groups [52, 53]. SCFAs are a subset of fatty acids produced by the gut microbiota through fermentation of partially digestible or nondigestible polysaccharides and play important roles in maintaining mucosal integrity, modulating metabolism, and regulating local and distal immune homeostasis [20, 72–74]. Compared to the URT, gut microbiomes showed more dispersion and heterogeneity among patients, as well as a greater distance to corresponding healthy controls, indicating a wider range of perturbed states in patient gut microbiota composition.

The human microbiome and its dynamics are important in modulating the host immune system; the recovery of microbiome homeostasis is also critical for the recovery of COVID-19 patients [75, 76]. SARS-CoV-2 infection can affect multiple organs; moreover, multi-faceted long-term symptoms have been reported for patients infected with SARS-CoV-2, with around 70–80% of patients showing at least one symptom 6 months after their discharge from the hospital [77]. The main symptoms were fatigue, muscle weakness, sleep disturbance, dyspnea, anxiety/depression, hair loss, loss of taste/smell, chest pain, and diarrhea [78]. Incidentally, studies have shown that alteration of the gut microbiota persists in a significant subset of patients with COVID-19 even after disease resolution and clearance of SARS-CoV-2 [22, 27]. One study found that gut microbiota richness was not restored to normal levels even up to 6 months after hospital discharge [79]; another recently revealed the association of gut microbiota with post-acute COVID-19 syndrome [80]. Thus far, there is accumulating evidence of respiratory tract and gut microbiota alterations as a result of SARS-CoV-2 infection, leading to the depletion of normal flora and enrichment of pathogenic species along with overall reduced microbiome diversity [23, 24, 81]. One study with a small sample size reported a synchronous transition of both URT and gut microbiome from early dysbiosis towards late more diverse status in mild COVID-19 patients during hospitalization [81]. In our study cohort, both URT and gut microbiota remained relatively stable during the study period, and no obvious trend of restoration was observed. Host- or environment-specific patterns of microbiome disruption, as well as impaired host immunity at different body sites could pose varying challenges to restoring microbiome and immunological homeostasis when recovering from COVID-19. Further investigation is needed to fully understand the role of the microbiome in host immunity against SARS-CoV-2 infection, as well as its relationship to the long-term effects post-COVID-19.

Along with changes in microbiome composition, different gene expression profiles that suggest functional changes were also observed in microbial communities of the URT and gut. An abundance of bacterial stress-response and toxin genes were detected in patients’ pharyngeal swabs and sputum. Genes related to the transport of diverse molecules were also enriched in patients’ microbiota, including components of antibiotic resistance-associated multi-drug efflux systems. Together with the discovery of reduced microbial diversity and expansion of a single microbe in the URT, this raises concerns regarding secondary infections and antimicrobial resistance in COVID-19 patients. In the gut microbiota of patients, there was a loss of genes related to fatty acid and carbohydrate metabolism, especially the depletion of SCFA-generation pathways, which is also consistent with the microbial compositional changes. Like the URT, patients’ gut microbiota also displayed elevated molecule transport and stress-response gene expression, further highlighting the stressful microenvironment associated with SARS-CoV-2 infection.

In summary, we revealed different types of the respiratory tract and gut microbiota alterations in COVID-19 patients, in terms of both microbial composition and function. We also did not observe any obvious trend of microbiome restoration during the study period, for both body sites sampled. Moreover, the compositional and functional profiling results further raise concerns about antibiotic resistance associated with the disease, which may further hinder the recovery of normal microbiota and leave long-term effects post-COVID-19. As such, more attention to potential antibiotic resistance and microbial homeostasis during clinical care of COVID-19 patients could offer additional insights for improving outcomes.

There are some limitations to this study. Due to the urgency and special situation of this disease, and pressures on clinical resources, sampling timepoints, and recordings of detailed clinical procedures were sometimes sacrificed to prioritize clinical care, resulting in missing samples at certain timepoints, or lack of sampling at baseline. Longer follow-up after patients’ recovery would also be more helpful for evaluating the relationship between alteration patterns and the restoration of microbiota in different body sites. Additional fecal samples from more symptomatically severe patients would also be beneficial to further elucidate the association of the gut microbiota and disease status; currently, this sample type is lacking due to sampling difficulties in the clinic. Since sputum samples may contain flora from both the upper and lower respiratory tract, they are therefore not a classical URT sample type. As such, we have used them mainly to supplement our findings from the pharyngeal swab samples. It is also extremely difficult to experimentally validate the findings of potential antibiotic resistance, stress response-, and toxin-related microbial pathways since the original samples are of limited quantity.

## Conclusions

We systematically assessed and compared the changes of microbiota from different body sites of COVID-19 patients and discovered distinguishing dysbiosis patterns between the respiratory tract and gut microbial communities. While there is a depletion of normal flora in both sample types, the gut microbiota is more sensitive to SARS-CoV-2 abundance and showed higher variability among patients. In terms of microbial function, gut microbiota show loss of carbohydrate and fatty acid metabolism, especially genes important for SCFA generation, while in the respiratory tract microbial community, stress response- and toxin-related genes are highly enriched and abundant. This study also revealed a potential problem of antimicrobial resistance in the clinical management of COVID-19 patients. While the prophylactic application of antibiotics is sometimes essential for the prevention and treatment of secondary infections, close monitoring and strategies for more precise use of antibiotics are urgently required in the ongoing SARS-CoV-2 pandemic.

## Supplementary Information

**Additional file 1: Figure S1.** Related to Fig. 2. Altered microbial composition associated with disease severity. (A) Comparison of alpha diversity between subjects with different disease severity in three sample types. (B) Differentially enriched genus associated with disease severity identified by LEfSe in sputum samples. (C) (D) (E) show differentially enriched species associated with severity in pharyngeal, sputum and faeces samples respectively. (F) Comparison of the abundance of patient-enriched genera in subjects with different disease severity in three sample types. **Figure S2.** Related to Fig. 2. Identification of *Halomonas* species in patient samples. (A) Distribution of sequence identity for classification of *Halomonas* species using Megablast. (B) Genomic similarity between Megahit assembled contigs and representative *Halomonas* species identified by Megablast. metaSpades assembly was shown in gray bar. Species reported in clinical environment were highlighted in yellow. Dashed line indicates 60% similarity. (C) Circular genomic comparison between Megahit assembly and representative *Halomonas* species with >60% of similarity. **Figure S3.** Related to Fig. 3. Comparison of the alteration patterns of microbial composition in upper respiratory tract and gut samples. PCoA analysis of the microbial composition in pharyngeal (A) and sputum (C) samples; and PERMANOVA test was applied to identify meta factors potentially associated with the microbial composition in pharyngeal (B) and sputum (D) samples from mild and moderate patients. (E) Distribution of the correlation coefficients between SARS-CoV-2 and representative genus in three sample types. **Figure S4.** Longitudinal assessment of the microbial composition in COVID-19 patients. The abundance of differential genera were shown in all time points for each patient during the study period; samples including pharyngeal (A), sputum (B) and faeces (C). **Figure S5.** Dynamics of Bray-Curtis distance in COVID-19 patients. The longitudinal trend of Bray-Curtis distance between patient samples to healthy controls were profiled for pharyngeal (A), sputum (B) and faeces (C).

**Additional file 2: Table S1.** Information of patients and samples included in this study.

**Additional file 3: Table S2.** Microbes detected in non-template controls.

## Data Availability

The sequencing data generated for this study have been uploaded to Genome Sequencing Archive (PRJCA002533).

## Abbreviations

URT: Upper respiratory tract
ACE2: Angiotensin-converting enzyme 2
NTCs: Non-template controls
PERMANOVA: Permutational multivariate analysis of variance
LEfSe: Linear discriminant analysis effect size
RNA: Ribonucleic acid
DNA: Deoxyribonucleic acid
SCFA: Short-chain fatty acid
PCR: Polymerase chain reaction
qPCR: Quantitative polymerase chain reaction
RT-PCR: Real-time polymerase chain reaction
RT-qPCR: Quantitative reverse transcription polymerase chain reaction
rRNA: Ribosomal RNA
rDNA: Ribosomal DNA
N10: Random decamer
dNTPs: Deoxyribonucleotide-tri-phosphates
T30VN: Oligo-dT, an oligomer of thymines
