# Supplementary figures and images for: Assessment of microbiota in the gut and upper respiratory tract associated with SARS-CoV-2 infection
Source: Microbiome. 2023 Mar 3;11:38. doi: 10.1186/s40168-022-01447-0 (PMC9982190; doi:10.1186/s40168-022-01447-0)

Fig S1

A

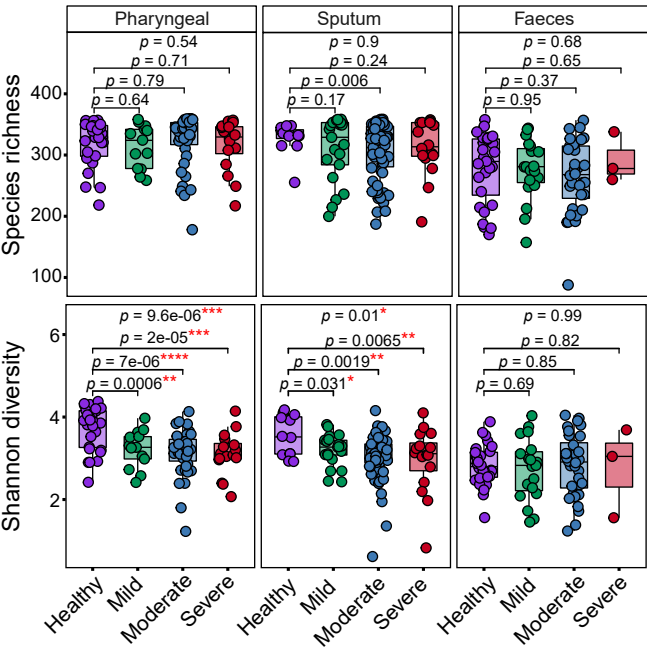

B

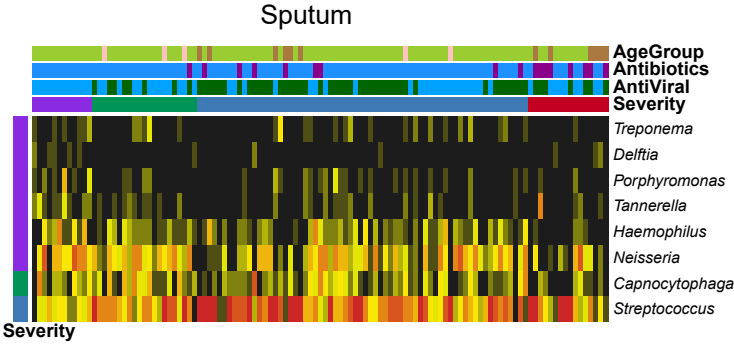

E

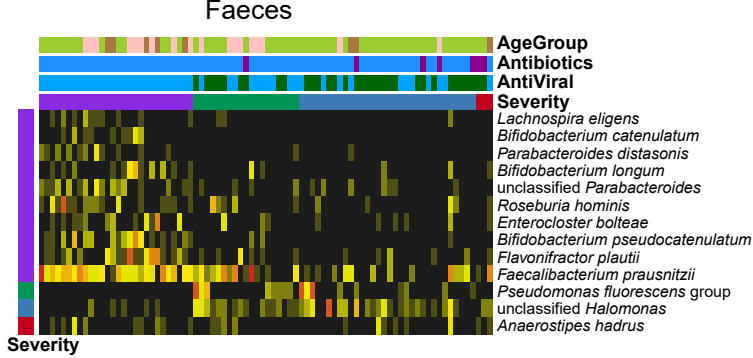

F

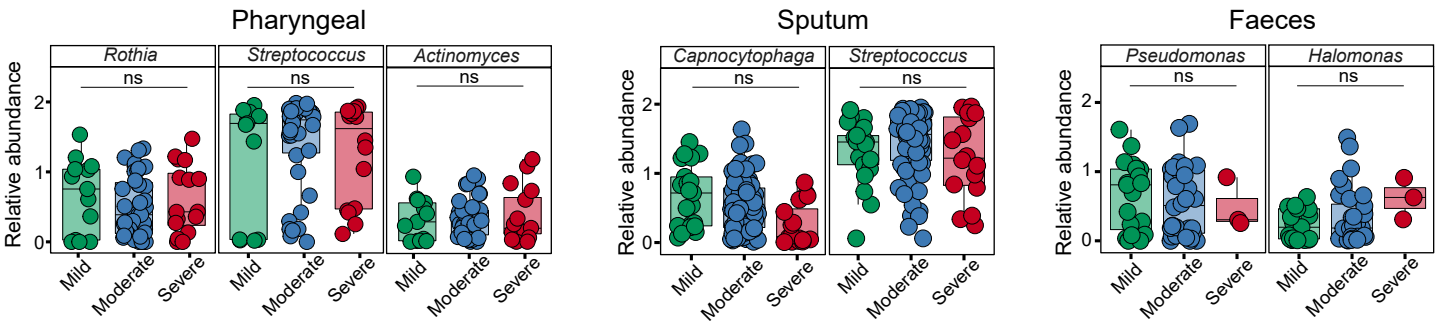

C

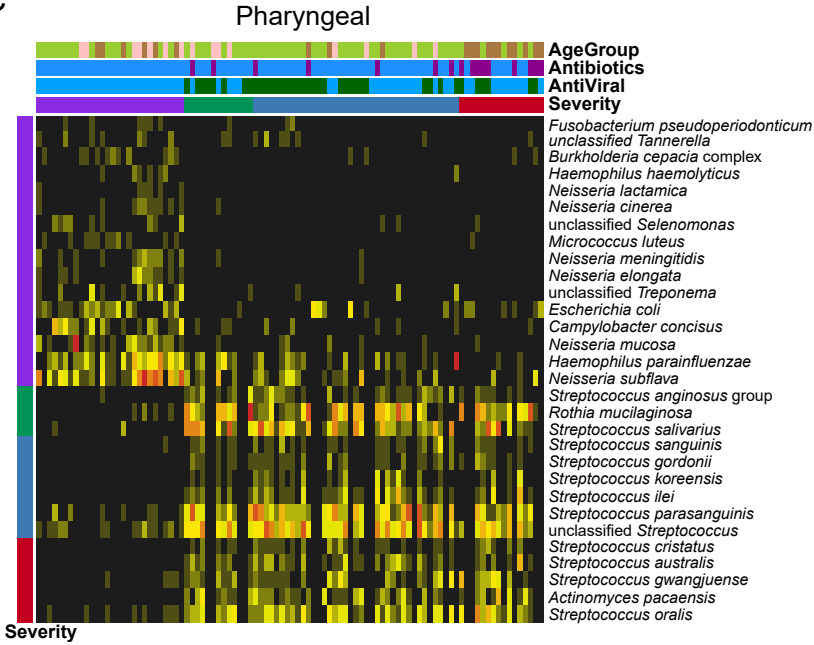

D

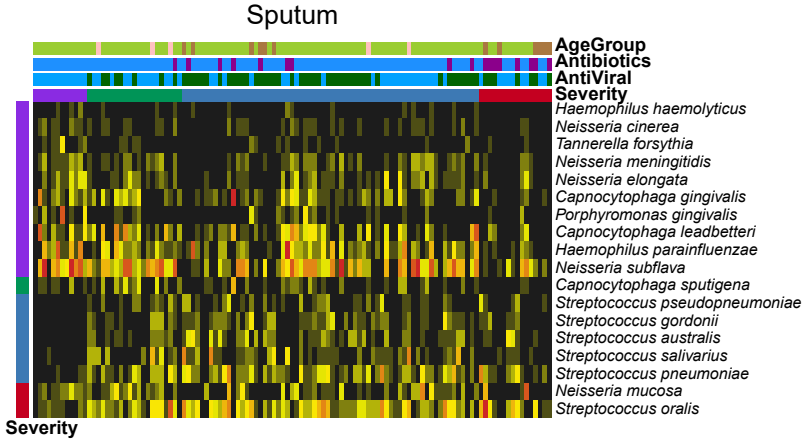

Fig S2

A

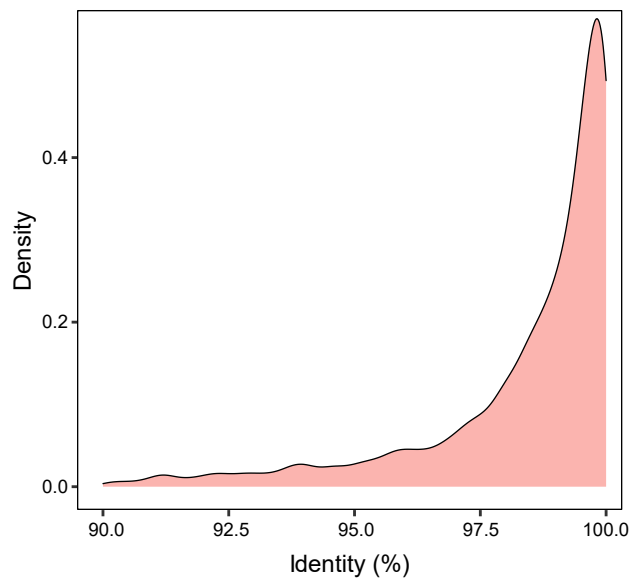

B

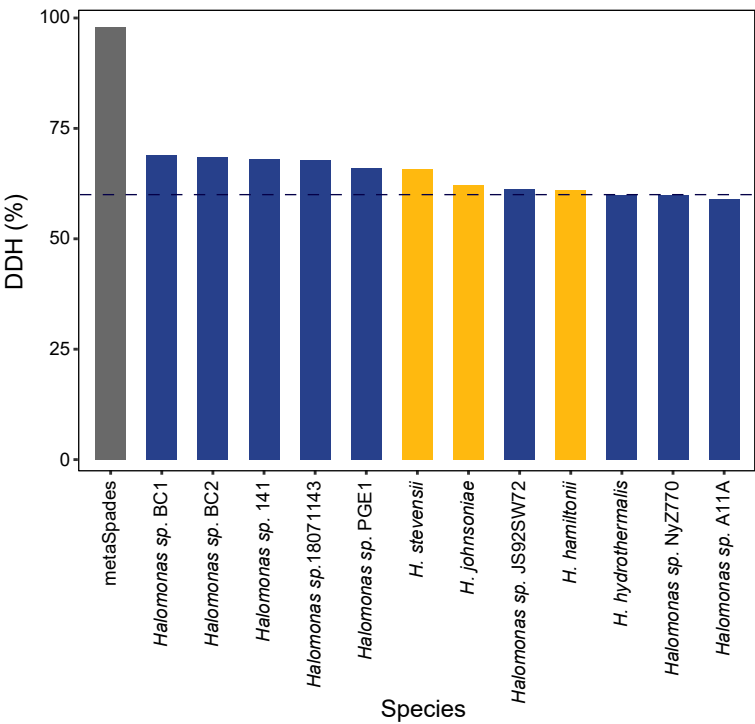

C

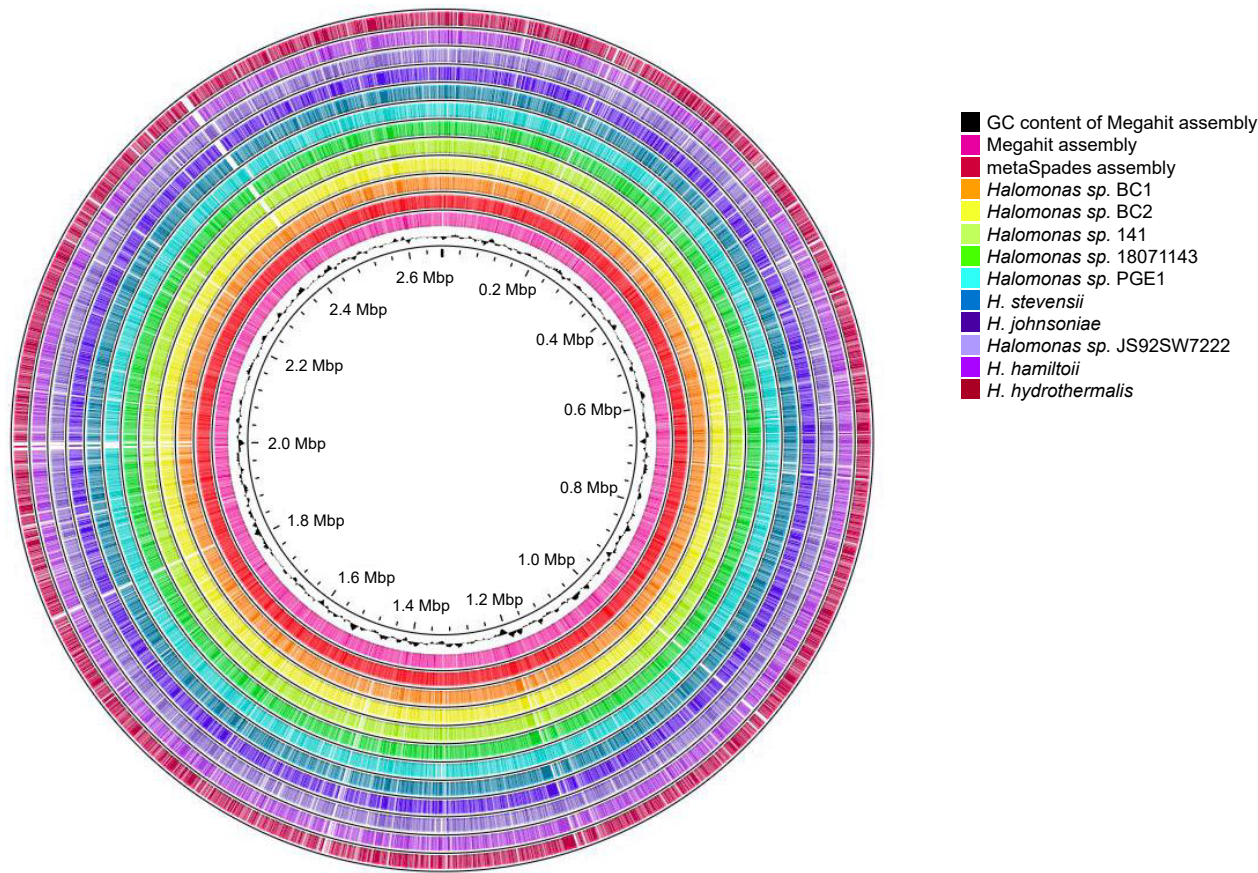

Fig S3

A

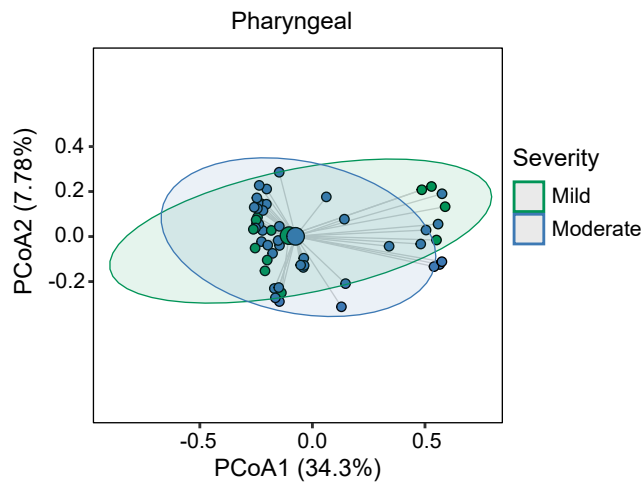

B

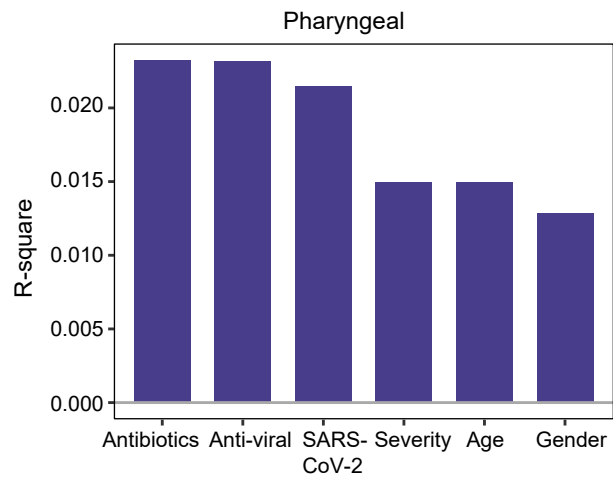

C

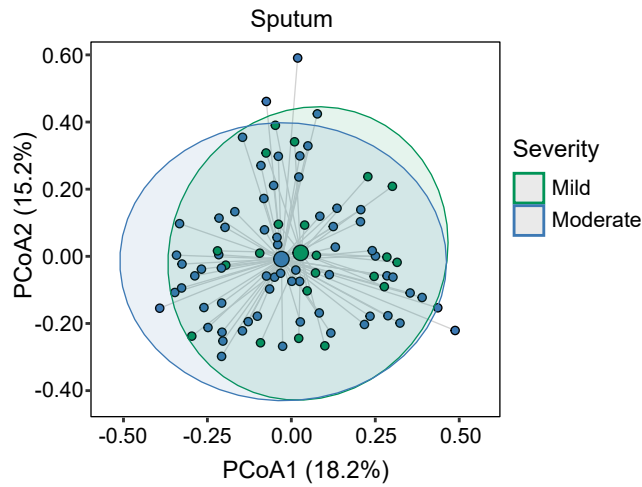

D

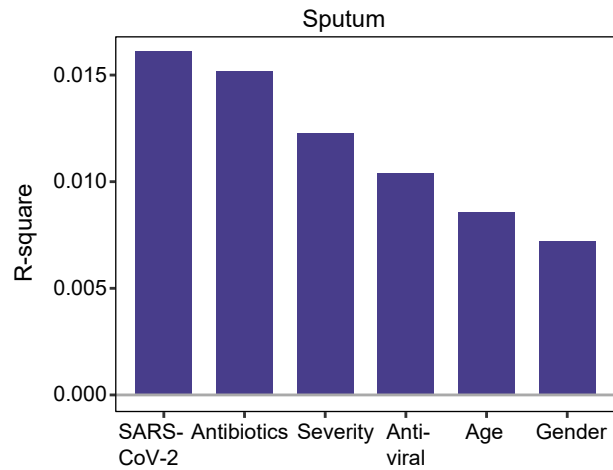

E

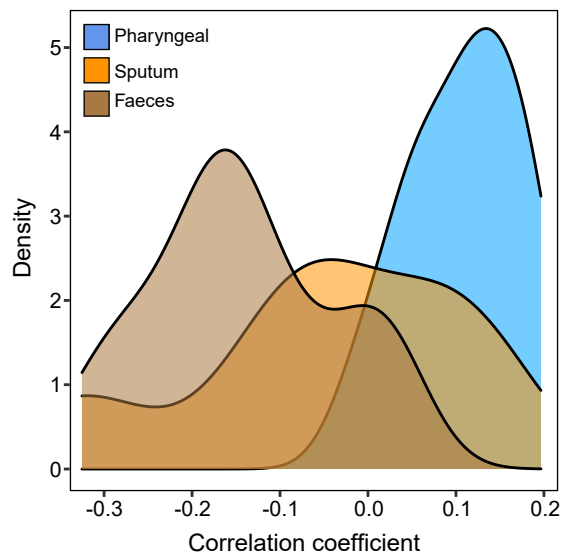

Fig S4

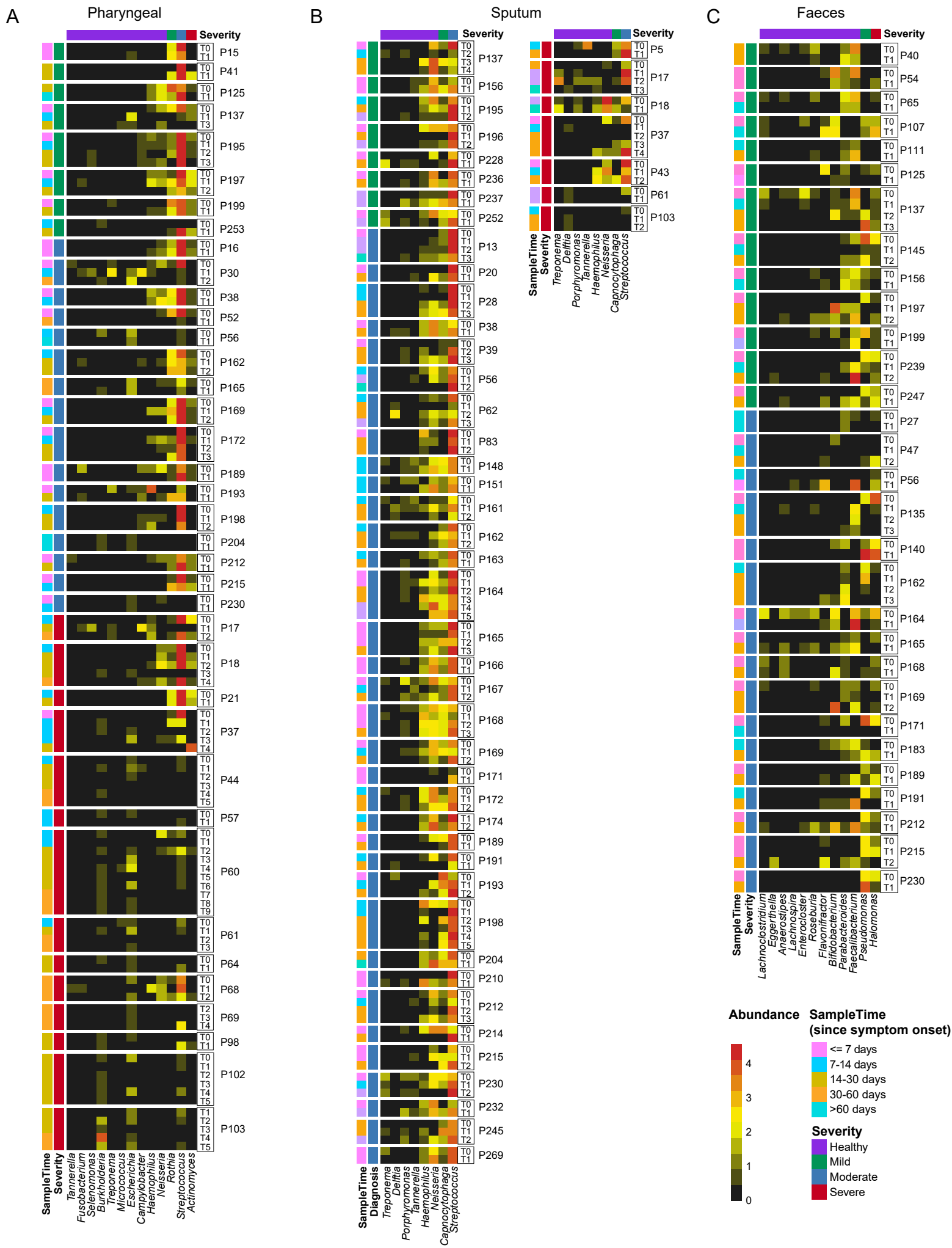

Fig S5

A

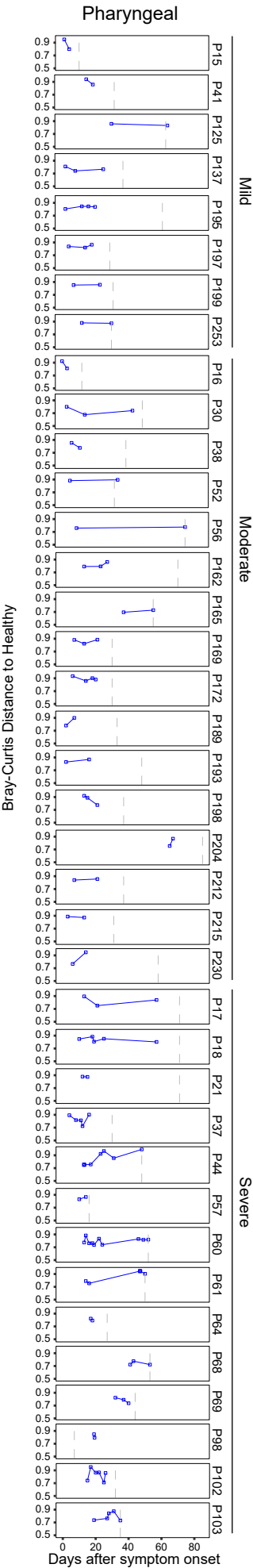

B

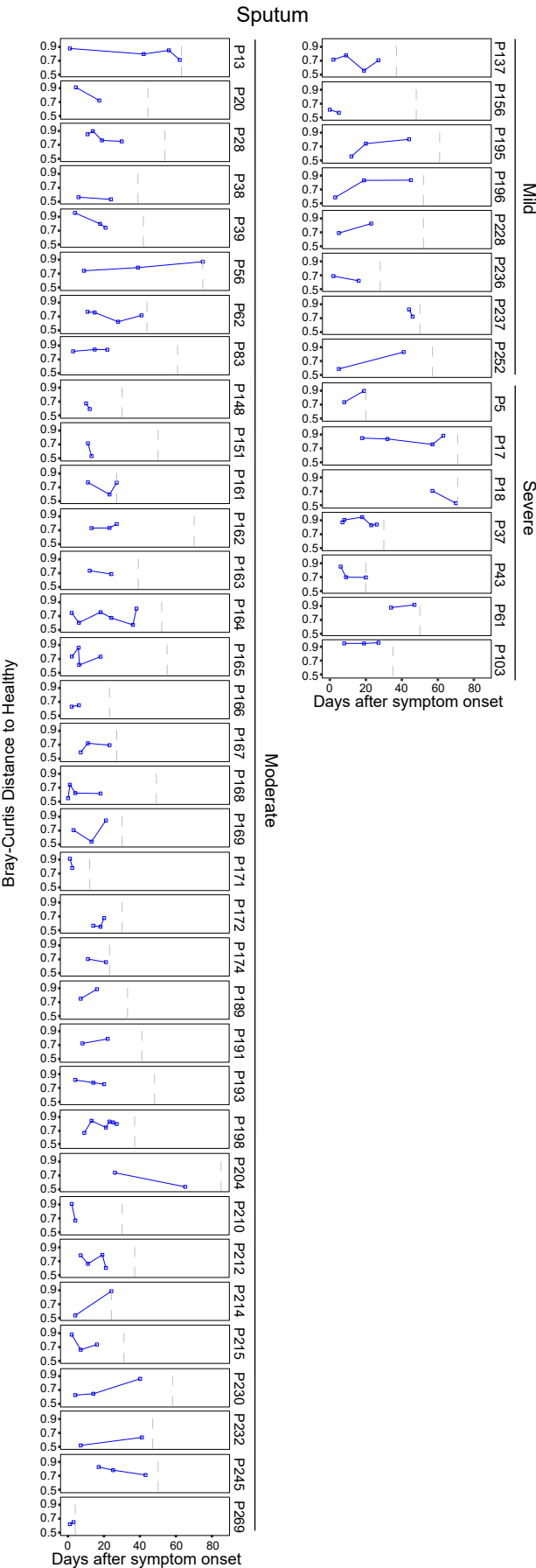

C

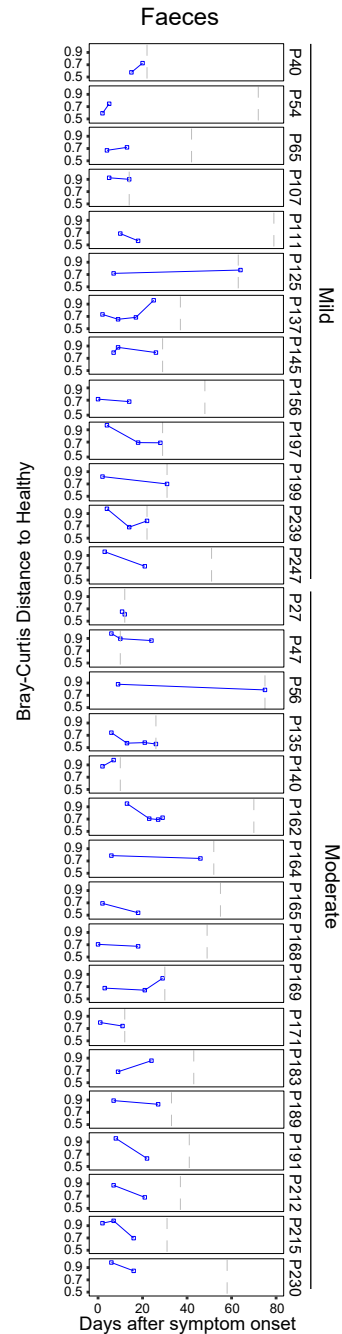

Supplement: Supplementary file 2 — Additional file 1: Figure S1. Related to Fig. 2. Altered microbial composition associated with disease severity. (A) Comparison of alpha diversity between subjects with different disease severity in three sample types. (B) Differentially enriched genus associated with disease severity identified by LEfSe in sputum samples. (C) (D) (E) show differentially enriched species associated with severity in pharyngeal, sputum and faeces samples respectively. (F) Comparison of the abundance of patient-enriched genera in subjects with different disease severity in three sample types. Figure S2. Related to Fig. 2. Identification of Halomonas species in patient samples. (A) Distribution of sequence identity for classification of Halomonas species using Megablast. (B) Genomic similarity between Megahit assembled contigs and representative Halomonas species identified by Megablast. metaSpades assembly was shown in gray bar. Species reported in clinical environment were highlighted in yellow. Dashed line indicates 60% similarity. (C) Circular genomic comparison between Megahit assembly and representative Halomonas species with >60% of similarity. Figure S3. Related to Fig. 3. Comparison of the alteration patterns of microbial composition in upper respiratory tract and gut samples. PCoA analysis of the microbial composition in pharyngeal (A) and sputum (C) samples; and PERMANOVA test was applied to identify meta factors potentially associated with the microbial composition in pharyngeal (B) and sputum (D) samples from mild and moderate patients. (E) Distribution of the correlation coefficients between SARS-CoV-2 and representative genus in three sample types. Figure S4. Longitudinal assessment of the microbial composition in COVID-19 patients. The abundance of differential genera were shown in all time points for each patient during the study period; samples including pharyngeal (A), sputum (B) and faeces (C). Figure S5. Dynamics of Bray-Curtis distance in COVID-19 patients. The lon [file 40168_2022_1447_MOESM1_ESM.pdf]
