# Supplementary material for: Assessment of microbiota in the gut and upper respiratory tract associated with SARS-CoV-2 infection
Source: Microbiome. 2023 Mar 3;11:38. doi: 10.1186/s40168-022-01447-0 (PMC9982190; doi:10.1186/s40168-022-01447-0)
Supplement: Supplementary file 3 — Additional file 2: Table S1. Information of patients and samples included in this study. [file 40168_2022_1447_MOESM2_ESM.pdf]

Table S1. Information of patients and samples included in this study

Sample	Patient	Group	SampleType	Gender	Age	Severity	RelativeDays	TimePoint	Antibiotics	Anti-viral	CtN	Raw	Nonhuman
DTcov-0311-2-sherry-m3	P8	Patient	Sputum	M	42	Moderate	15	T0	NA	Yes	32.425	2468936	494070
DTcov-0311-3-sherry-m3	P20	Patient	Sputum	F	63	Moderate	4	T0	No	Yes	20.524	39092827	8023735
DTcov-0311-4-sherry-m3	P28	Patient	Sputum	F	33	Moderate	11	T0	Yes	Yes	NA	5013516	787922
DTcov-0311-5-sherry-m3	P43	Patient	Sputum	M	35	Severe	9	T1	Yes	Yes	32.332	31683662	2361502
DTcov-0311-6-sherry-m3	P83	Patient	Sputum	M	63	Moderate	3	T0	No	Yes	18.637	39139616	4297298
DTcov-0311-7-sherry-m3	P59	Patient	Sputum	M	58	Severe	13	T0	Yes	No	30.119	26331498	3490992
DTcov-0311-8-sherry-m3	P59	Patient	Pharyngeal	M	58	Severe	13	T0	Yes	Yes	NA	23484173	4366334
DTcov-0311-9-sherry-m3	P44	Patient	Pharyngeal	M	82	Severe	13	T0	No	No	18.752	10092707	1513285
DTcov-0311-10-sherry-m3	P68	Patient	Pharyngeal	M	57	Severe	43	T1	NA	No	NA	1979521	292498
DTcov-0311-11-sherry-m3	P38	Patient	Pharyngeal	F	32	Moderate	11	T1	No	Yes	NA	20857619	4936618
DTcov-0311-12-sherry-m3	P40	Patient	Pharyngeal	F	3	Mild	15	T0	No	Yes	26.622	19935080	2872201
DTcov-0311-13-sherry-m3	P41	Patient	Pharyngeal	F	5	Mild	15	T0	No	Yes	31.066	3655972	995977
DTcov-0311-14-sherry-m3	P60	Patient	Pharyngeal	F	78	Severe	14	T1	Yes	No	27.626	10463234	1012355
DTcov-0311-15-sherry-m3	P61	Patient	Pharyngeal	M	82	Severe	14	T0	NA	No	24.066	1487902	132205
DTcov-0311-16-sherry-m3	P64	Patient	Pharyngeal	M	69	Severe	18	T1	No	No	30.239	9206968	919591
DTcov-0311-17-sherry-m3	P57	Patient	Pharyngeal	M	88	Severe	14	T1	NA	Yes	26.732	15710532	3373926
DTcov-0314-18-sherry-m1	P145	Patient	Faeces	M	24	Mild	7	T0	No	Yes	21.764	17808417	273574
DTcov-0314-19-sherry-m1	P145	Patient	Faeces	M	24	Mild	9	T1	No	Yes	NA	17887577	1126424
DTcov-0314-20-sherry-m1	P142	Patient	Faeces	F	22	Moderate	13	T0	No	No	99.99	18411449	226583
DTcov-0314-21-sherry-m1	P182	Patient	Faeces	F	11	Mild	1	T0	No	No	23.339	11406774	384049
DTcov-0314-22-sherry-m1	P143	Patient	Faeces	F	40	Moderate	4	T0	No	Yes	99.99	18898931	467215
DTcov-0314-23-sherry-m1	P178	Patient	Sputum	F	55	Moderate	4	T0	No	Yes	35.723	18211079	2612059
DTcov-0314-24-sherry-m1	P140	Patient	Faeces	M	21	Moderate	7	T1	No	Yes	99.99	16323223	14482545
DTcov-0314-25-sherry-m1	P137	Patient	Pharyngeal	M	30	Mild	2	T0	No	Yes	99.99	17148889	3314711
DTcov-0314-26-sherry-m1	P137	Patient	Sputum	M	30	Mild	2	T0	No	Yes	28.719	20752385	1924866
DTcov-0314-27-sherry-m1	P171	Patient	Faeces	F	47	Moderate	1	T0	No	Yes	99.99	17401381	309059
DTcov-0314-28-sherry-m1	P171	Patient	Sputum	F	47	Moderate	1	T0	No	Yes	99.99	11100605	1187883
DTcov-0314-29-sherry-m1	P181	Patient	Sputum	F	18	Mild	5	T0	No	No	NA	16597896	1480193
DTcov-0314-30-sherry-m1	P182	Patient	Sputum	F	11	Mild	4	T0	No	No	99.99	18187826	1834920
DTcov-0314-31-sherry-m1	P146	Patient	Faeces	F	24	Mild	4	T0	No	Yes	99.99	24160688	1035181
DTcov-0314-32-sherry-m1	P140	Patient	Sputum	M	21	Moderate	2	T0	No	Yes	34.604	1427614	338880
DTcov-0318-034	P13	Patient	Sputum	M	33	Moderate	1	T0	No	No	18.73	5300922	835001
DTcov-0318-035	P17	Patient	Sputum	F	72	Severe	18	T0	No	Yes	30.655	2426574	619841
DTcov-0318-036	P28	Patient	Sputum	F	33	Moderate	14	T1	Yes	Yes	34.636	55506497	5513710
DTcov-0318-037	P44	Patient	Pharyngeal	M	82	Severe	17	T1	No	No	28.519	2202567	184865
DTcov-0318-039	P60	Patient	Pharyngeal	F	78	Severe	18	T3	Yes	Yes	32.394	1987593	263095
DTcov-0318-040	P61	Patient	Pharyngeal	M	82	Severe	16	T1	NA	No	31.045	2455378	286824
DTcov-0318-041	P68	Patient	Pharyngeal	M	57	Severe	41	T0	NA	No	26.506	2974091	263758
DTcov-0318-043	P71	Patient	Pharyngeal	F	81	Severe	2	T0	NA	Yes	29.721	10642295	1419615
DTcov-0318-044	P5	Patient	Pharyngeal	M	49	Severe	8	T0	Yes	Yes	29.815	7898876	1579626
DTcov-0318-045	P8	Patient	Pharyngeal	M	42	Moderate	7	T0	NA	Yes	27.225	9838150	1952079
DTcov-0318-046	P9	Patient	Pharyngeal	M	41	Moderate	5	T0	No	Yes	25.797	15496315	2672772
DTcov-0318-047	P14	Patient	Pharyngeal	M	47	Moderate	6	T0	No	Yes	27.987	10034335	1278475
DTcov-0318-048	P15	Patient	Pharyngeal	F	0.8	Mild	1	T0	Yes	No	28.583	12286185	2318133
DTcov-0318-049	P16	Patient	Pharyngeal	F	36	Moderate	3	T1	No	Yes	29.394	14986090	2696632
DiTan-0323-50	P169	Patient	Pharyngeal	M	17	Moderate	7	T0	No	Yes	23.621	16097611	3220837
DiTan-0323-51	P172	Patient	Pharyngeal	F	44	Moderate	6	T0	No	Yes	24.741	15870299	4544027
DiTan-0323-52	P179	Patient	Pharyngeal	F	22	Moderate	2	T0	No	Yes	27.759	18511658	3064879
DiTan-0323-53	P194	Patient	Pharyngeal	F	23	Moderate	3	T0	No	Yes	25.47	18529912	2732709
DiTan-0323-54	P189	Patient	Pharyngeal	F	40	Moderate	2	T0	No	Yes	21.22	19235081	3265342
DiTan-0323-55	P195	Patient	Pharyngeal	M	28	Mild	2	T0	No	Yes	19.545	18952231	3837017
DiTan-0323-56	P191	Patient	Pharyngeal	F	36	Moderate	4	T0	No	Yes	26.877	18384236	3581067
DiTan-0323-57	P197	Patient	Pharyngeal	F	28	Mild	4	T0	No	Yes	21.964	17374462	3346679
DiTan-0323-58	P153	Patient	Faeces	F	22	Mild	4	T0	No	Yes	18.246	10868727	638484
DiTan-0323-59	P199	Patient	Faeces	F	32	Mild	2	T0	No	Yes	27.629	3448702	270289
DiTan-0323-60	P148	Patient	Sputum	F	13	Moderate	12	T1	No	Yes	NA	14338327	2339720
DiTan-0323-61	P162	Patient	Sputum	M	40	Moderate	13	T0	No	No	30.537	6299899	939891
DiTan-0323-62	P159	Patient	Sputum	F	47	Moderate	5	T0	Yes	Yes	24.931	9033022	1399198
DiTan-0323-63	P156	Patient	Sputum	M	42	Mild	5	T1	No	Yes	31.367	11884154	1141437
DiTan-0323-64	P218	Patient	Sputum	M	37	Moderate	4	T0	No	No	35.25	11106803	1121648
DiTan-0323-65	P186	Patient	Sputum	F	22	Moderate	3	T0	No	Yes	29.667	12187503	1167864
DiTan-0323-66	P193	Patient	Sputum	M	41	Moderate	4	T0	Yes	Yes	30.363	11039165	2729856
DiTan-0323-67	P183	Patient	Sputum	M	49	Moderate	8	T0	No	Yes	24.188	11750513	1612208
DiTan-0323-68	P184	Patient	Sputum	F	48	Moderate	3	T0	No	Yes	99.99	14247171	2602295
DiTan-0323-69	P196	Patient	Sputum	F	27	Mild	3	T0	No	Yes	28.05	14067814	3831180
DiTan-0323-70	P220	Patient	Sputum	M	27	Moderate	7	T0	No	Yes	24.94	12026226	1187881
DiTan-0323-71	P221	Patient	Sputum	F	69	Severe	9	T0	No	Yes	99.99	15473073	2455028
DiTan-0323-72	P81	Patient	Pharyngeal	M	21	Moderate	4	T0	No	Yes	29.415	5273745	1036507
DiTan-0323-73	P37	Patient	Pharyngeal	F	86	Severe	4	T0	No	Yes	29.394	10236853	1503578
DiTan-0323-74	P77	Patient	Pharyngeal	F	33	Moderate	16	T0	No	Yes	24.688	5491391	1496732
DiTan-0323-75	P39	Patient	Pharyngeal	F	64	Moderate	4	T0	Yes	Yes	20.868	6337724	849815
DiTan-0323-76	P130	Patient	Faeces	M	3	Moderate	29	T0	No	No	18.261	2494436	104031

DiTan-0323-77	P111	Patient	Faeces	F	3	Mild	18	T1	No	No	13.836	20854263	664662
DiTan-0323-78	P102	Patient	Pharyngeal	M	67	Severe	17	T1	No	No	32.419	10815767	1170985
DiTan-0323-79	P101	Patient	Sputum	M	69	Severe	17	T0	NA	No	22.379	8015652	1076912
DiTan-0323-81	P67	Patient	Faeces	M	46	Moderate	12	T0	No	Yes	22.403	1755681	107785
DTcov-0331-082	P5	Patient	Sputum	M	49	Severe	8	T0	Yes	Yes	99.99	33461507	3212433
DTcov-0331-083	P30	Patient	Sputum	M	73	Moderate	3	T0	No	No	20.28	3941854	424059
DTcov-0331-084	P32	Patient	Sputum	F	20	Moderate	6	T0	No	Yes	19.822	5604600	602026
DTcov-0331-085	P39	Patient	Sputum	F	64	Moderate	4	T0	Yes	Yes	23.591	18024622	1683305
DTcov-0331-086	P35	Patient	Sputum	F	61	Moderate	7	T0	No	Yes	21.197	22841425	1998054
DTcov-0331-087	P34	Patient	Sputum	M	37	Moderate	5	T0	No	Yes	16.338	5334289	741293
DTcov-0331-088	P33	Patient	Sputum	F	76	Moderate	14	T0	No	Yes	21.365	16051454	2370305
DTcov-0331-089	P61	Patient	Pharyngeal	M	82	Severe	47	T2	NA	No	24.141	90048297	6824623
DTcov-0331-090	P44	Patient	Pharyngeal	M	82	Severe	48	T5	No	No	99.99	146924851	6406253
DTcov-0331-091	P60	Patient	Sputum	F	78	Severe	49	T0	Yes	No	99.99	13761119	1023554
DTcov-0331-092	P69	Patient	Pharyngeal	F	84	Severe	40	T4	No	No	27.458	19599993	1068266
DTcov-0331-093	P18	Patient	Pharyngeal	M	74	Severe	10	T0	No	No	28.666	48439555	11315945
DTcov-0331-094	P18	Patient	Pharyngeal	M	74	Severe	57	T4	No	Yes	26.734	29001384	1801276
DTcov-0331-095	P17	Patient	Pharyngeal	F	72	Severe	13	T0	No	No	28.706	36614283	9966114
DTcov-0331-096	P17	Patient	Pharyngeal	F	72	Severe	57	T2	No	No	32.827	17693004	2728927
DTcov-0331-097	P151	Patient	Sputum	F	41	Moderate	11	T0	No	Yes	25.819	56430615	6572919
DTcov-0331-098	P187	Patient	Sputum	F	28	Mild	6	T0	No	Yes	30.605	43271765	4889290
DTcov-0331-099	P228	Patient	Sputum	F	22	Mild	5	T0	No	No	19.873	919744	156659
DTcov-0331-100	P235	Patient	Sputum	F	25	Moderate	3	T0	No	No	25.275	25763657	3989410
DTcov-0331-101	P239	Patient	Sputum	M	27	Mild	4	T0	No	Yes	23.39	2076650	254588
DTcov-0331-102	P13	Patient	Sputum	M	33	Moderate	42	T1	No	No	31.095	38845063	7315003
DTcov-0331-103	P260	Patient	Pharyngeal	M	52	Severe	8	T0	No	No	22.68	1687437	122912
DTcov-0331-104	P192	Patient	Sputum	M	19	Mild	9	T0	No	Yes	35.361	38187354	4444877
DTcov-0331-105	P198	Patient	Sputum	F	21	Moderate	9	T0	No	No	24.456	48833774	3809382
DTcov-0331-106	P207	Patient	Pharyngeal	M	22	Mild	8	T0	No	Yes	27.87	31165564	3796830
DTcov-0331-107	P210	Patient	Sputum	F	24	Moderate	4	T1	No	Yes	31.538	36865795	7566270
DTcov-0331-108	P176	Patient	Sputum	F	37	Moderate	15	T0	No	No	27.777	16011605	2506259
DTcov-0331-109	P214	Patient	Sputum	F	52	Moderate	4	T0	No	Yes	25.718	25261308	4562656
DTcov-0331-110	P244	Patient	Sputum	M	26	Moderate	9	T0	No	No	32.103	27838272	2765972
DTcov-0331-111	P247	Patient	Sputum	F	23	Mild	3	T0	No	No	18.537	1447205	208906
DTcov-0331-112	P252	Patient	Sputum	F	28	Mild	5	T0	No	No	29.574	21696089	2094460
DTcov-0331-113	P253	Patient	Pharyngeal	M	4	Mild	12	T0	No	Yes	31.385	28962515	1265738
DTcov-0403-114	P137	Patient	Faeces	M	30	Mild	2	T0	No	Yes	99.99	119481155	4379589
DTcov-0403-115	P137	Patient	Faeces	M	30	Mild	9	T1	No	Yes	32.295	96609388	3290886
DTcov-0403-116	P137	Patient	Faeces	M	30	Mild	17	T2	No	Yes	25.366	876043	265110
DTcov-0403-117	P137	Patient	Sputum	M	30	Mild	19	T3	No	Yes	29.427	91212740	11627100
DTcov-0403-118	P137	Patient	Pharyngeal	M	30	Mild	25	T3	No	Yes	28.954	43345920	2788780
DTcov-0403-119	P137	Patient	Faeces	M	30	Mild	25	T3	No	Yes	26.849	1241104	100154
DTcov-0403-120	P137	Patient	Sputum	M	30	Mild	27	T4	No	Yes	32.891	68028655	8401638
DTcov-0403-121	P141	Patient	Sputum	M	22	Moderate	21	T0	No	Yes	30.774	61048022	9471265
DTcov-0403-122	P111	Patient	Faeces	F	3	Mild	10	T0	No	No	30.464	62531557	3642880
DTcov-0403-123	P112	Patient	Faeces	M	29	Moderate	13	T0	No	No	26.061	18038879	727333
DTcov-0403-124	P204	Patient	Faeces	F	36	Moderate	56	T0	No	No	32.452	2238395	275143
DTcov-0403-126	P18	Patient	Pharyngeal	M	74	Severe	19	T2	No	No	32.643	51948648	8276353
DTcov-0403-127	P110	Patient	Faeces	F	29	Moderate	16	T0	No	Yes	99.99	2135296	197038
DTcov-0410-128	P17	Patient	Sputum	F	72	Severe	32	T1	No	Yes	25.071	23954438	2512033
DTcov-0403-129	P164	Patient	Sputum	M	50	Moderate	18	T2	No	Yes	35.284	49344464	7515873
DTcov-0403-130	P135	Patient	Faeces	F	58	Moderate	6	T0	No	Yes	29.103	22851770	1115723
DTcov-0403-131	P135	Patient	Faeces	F	58	Moderate	26	T3	No	Yes	31.58	47593322	4618059
DTcov-0403-132	P139	Patient	Faeces	M	32	Moderate	4	T0	No	Yes	29.039	46294428	2046268
DTcov-0403-133	P165	Patient	Sputum	M	33	Moderate	2	T0	No	Yes	27.769	17227311	2474699
DTcov-0403-134	P165	Patient	Sputum	M	33	Moderate	18	T3	No	Yes	33.245	51539127	8391812
DTcov-0403-135	P167	Patient	Sputum	F	40	Moderate	7	T0	No	Yes	31.132	50776685	6897099
DTcov-0403-136	P167	Patient	Sputum	F	40	Moderate	23	T2	No	Yes	35.126	38758657	8093466
DTcov-0403-137	P168	Patient	Sputum	F	51	Moderate	0	T0	No	Yes	32.621	45806910	6657755
DTcov-0403-138	P175	Patient	Sputum	M	58	Moderate	2	T0	No	Yes	29.751	44299494	6548583
DTcov-0403-139	P251	Patient	Pharyngeal	M	28	Mild	20	T0	No	No	28.471	14832722	931075
DTcov-0403-140	P269	Patient	Sputum	M	21	Moderate	3	T1	No	Yes	33.259	56780345	7116009
DTcov-0403-141	P272	Patient	Sputum	F	24	Moderate	9	T0	No	Yes	32.555	54516295	6665294
DTcov-0403-142	P273	Patient	Sputum	F	23	Moderate	2	T0	No	No	31.948	53195571	8062230
DTcov-0410-143	P164	Patient	Sputum	M	50	Moderate	2	T0	No	Yes	25.011	20099624	1802892
DTcov-0410-144	P154	Patient	Sputum	F	23	Moderate	8	T0	No	Yes	23.409	3564424	170456
DTcov-0410-145	P150	Patient	Faeces	M	6	Moderate	11	T0	No	No	30.784	3694592	837066
DTcov-0410-146	P65	Patient	Faeces	M	6	Mild	4	T0	No	No	23.91	1909718	130696
DTcov-0410-147	P65	Patient	Sputum	M	6	Mild	41	T0	No	No	23.389	3497422	271297
DTcov-0410-148	P54	Patient	Pharyngeal	M	7	Mild	1	T0	No	No	31.632	65982369	10604568
DTcov-0410-149	P125	Patient	Faeces	M	9	Mild	7	T0	No	Yes	28.399	59348449	4498775
DTcov-0410-150	P31	Patient	Sputum	M	37	Moderate	42	T0	No	Yes	29.63	49972286	3665622
DTcov-0410-151	P21	Patient	Pharyngeal	F	62	Severe	12	T0	No	No	32.683	53404983	8923027
DTcov-0410-152	P157	Patient	Faeces	M	23	Mild	4	T0	Yes	Yes	30.911	44934648	2350403

DTcov-0410-153	P161	Patient	Sputum	M	40	Moderate	11	T0	No	Yes	22.787	3043305	364425
DTcov-0410-154	P53	Patient	Pharyngeal	F	37	Moderate	4	T0	No	Yes	24.242	1100444	221335
DTcov-0410-155	P56	Patient	Pharyngeal	M	59	Moderate	9	T0	No	Yes	30.127	5820138	508986
DTcov-0410-156	P38	Patient	Sputum	F	32	Moderate	6	T0	No	Yes	99.99	36599100	13150862
DTcov-0410-157	P48	Patient	Sputum	M	37	Severe	7	T0	Yes	Yes	29.348	2108418	1359056
DTcov-0410-158	P18	Patient	Pharyngeal	M	74	Severe	18	T1	No	No	32.608	127127421	18301961
DTcov-0410-159	P67	Patient	Sputum	M	46	Moderate	4	T0	No	Yes	30.314	600106	287516
DTcov-0410-160	P56	Patient	Sputum	M	59	Moderate	9	T0	No	Yes	29.508	60664374	9049190
DTcov-0410-161	P32	Patient	Pharyngeal	F	20	Moderate	6	T0	No	Yes	29.607	4201157	713060
DTcov-0410-162	P30	Patient	Pharyngeal	M	73	Moderate	3	T0	No	No	29.603	30600718	3791746
DTcov-0410-163	P32	Patient	Faeces	F	20	Moderate	6	T0	No	Yes	29.863	20511133	1257081
DTcov-0410-164	P30	Patient	Faeces	M	73	Moderate	3	T0	No	No	99.99	24104564	1247547
DTcov-0410-165	P39	Patient	Faeces	F	64	Moderate	4	T0	Yes	Yes	30.861	2000579	178249
DTcov-0410-166	P62	Patient	Sputum	M	52	Moderate	11	T0	No	Yes	99.99	26392214	3202830
DTcov-0410-167	P52	Patient	Pharyngeal	F	2	Moderate	5	T0	No	No	28.668	36444346	9913749
DTcov-0410-168	P39	Patient	Sputum	F	64	Moderate	21	T3	Yes	Yes	36.758	3315967	1155851
DTcov-0410-169	P44	Patient	Pharyngeal	M	82	Severe	23	T2	No	No	29.463	657575	107793
DTcov-0410-170	P37	Patient	Sputum	F	86	Severe	26	T4	No	No	32.847	47197271	6999442
DTcov-0410-172	P28	Patient	Sputum	F	33	Moderate	30	T3	Yes	Yes	32.458	46531975	5000018
DTcov-0410-173	P38	Patient	Sputum	F	32	Moderate	24	T1	No	Yes	30.817	5167998	1047500
DTcov-0410-174	P102	Patient	Sputum	M	67	Severe	32	T0	No	No	30.051	11735414	2646320
DTcov-0410-175	P56	Patient	Sputum	M	59	Moderate	39	T1	No	No	99.99	45450029	7324637
DTcov-0410-176	P62	Patient	Sputum	M	52	Moderate	41	T3	Yes	Yes	99.99	35366495	5188979
DTcov-0410-177	P201	Patient	Sputum	F	76	Severe	5	T0	NA	No	29.54	4080078	343623
DTcov-0410-178	P168	Patient	Sputum	F	51	Moderate	18	T3	No	Yes	29.722	25393435	5006912
DTcov-0410-179	P174	Patient	Sputum	M	30	Moderate	21	T2	No	Yes	34.057	18535783	2321837
DTcov-0410-180	P53	Patient	Faeces	F	37	Moderate	47	T0	No	No	32.49	28253580	1001601
DTcov-0410-181	P61	Patient	Pharyngeal	M	82	Severe	50	T3	NA	No	29.557	8753533	578560
DTcov-0410-182	P103	Patient	Pharyngeal	M	78	Severe	31	T4	Yes	No	30.625	14423027	624906
DTcov-0410-183	P17	Patient	Sputum	F	72	Severe	57	T2	No	No	35.043	31024516	2486586
DTcov-0410-184	P13	Patient	Pharyngeal	M	33	Moderate	56	T0	No	No	32.229	28167697	6152701
DTcov-0410-185	P13	Patient	Sputum	M	33	Moderate	56	T2	No	No	31.615	32399243	10373292
DTcov-0410-186	P15	Patient	Pharyngeal	F	0.8	Mild	4	T1	Yes	No	29.242	2690386	343916
DTcov-0410-187	P16	Patient	Pharyngeal	F	36	Moderate	0	T0	No	Yes	31.133	63702159	7019976
DTcov-0410-188	P38	Patient	Pharyngeal	F	32	Moderate	6	T0	No	Yes	30.885	26841204	4504548
DTcov-0410-189	P40	Patient	Faeces	F	3	Mild	15	T0	No	Yes	24.115	55864509	1873722
DTcov-0410-190	P55	Patient	Pharyngeal	F	56	Moderate	12	T0	No	Yes	25.468	3421502	219739
DTcov-0410-191	P54	Patient	Faeces	M	7	Mild	5	T1	No	No	30.271	46713292	3296151
DTcov-0410-192	P40	Patient	Faeces	F	3	Mild	20	T1	No	Yes	32.134	47469542	1901991
DTcov-0410-193	P41	Patient	Pharyngeal	F	5	Mild	19	T1	No	Yes	29.096	59688959	13047108
DTcov-0410-194	P41	Patient	Sputum	F	5	Mild	19	T0	No	Yes	32.23	37441392	15221187
DTcov-0410-195	P41	Patient	Faeces	F	5	Mild	20	T0	No	Yes	28.019	39710104	1308850
DTcov-0410-196	P54	Patient	Faeces	M	7	Mild	2	T0	No	No	32.545	35539056	1125355
DTcov-0410-197	P56	Patient	Faeces	M	59	Moderate	9	T0	No	Yes	35.761	36057631	1542288
DTcov-0410-198	P166	Patient	Sputum	M	51	Moderate	2	T0	No	Yes	30.887	33069271	5359813
DTcov-0410-199	P166	Patient	Faeces	M	51	Moderate	2	T0	No	Yes	32.825	39375447	1316657
DTcov-0410-200	P165	Patient	Faeces	M	33	Moderate	2	T0	No	Yes	35.406	38107459	1120241
DTcov-0410-201	P168	Patient	Faeces	F	51	Moderate	0	T0	No	Yes	30.703	45095919	1374169
DTcov-0410-202	P140	Patient	Faeces	M	21	Moderate	2	T0	No	Yes	30.716	44813797	2811087
DTcov-0410-203	P163	Patient	Sputum	M	44	Moderate	12	T0	No	Yes	32.07	33466410	5347279
DTcov-0410-204	P163	Patient	Faeces	M	44	Moderate	12	T0	No	Yes	34.953	35107157	1182415
DTcov-0410-205	P165	Patient	Faeces	M	33	Moderate	18	T1	No	Yes	35.726	3136231	364485
DTcov-0410-206	P171	Patient	Faeces	F	47	Moderate	11	T1	No	Yes	35.757	21706506	2089698
DTcov-0410-207	P151	Patient	Faeces	F	41	Moderate	4	T0	No	Yes	36.13	30398875	911768
DTcov-0410-208	P163	Patient	Pharyngeal	M	44	Moderate	24	T0	No	Yes	29.658	37405636	4009063
DTcov-0410-209	P163	Patient	Sputum	M	44	Moderate	24	T1	No	Yes	30.667	39407289	4620574
DTcov-0410-210	P148	Patient	Sputum	F	14	Moderate	10	T0	No	Yes	32.989	53315330	3942074
DTcov-0410-211	P151	Patient	Sputum	F	41	Moderate	13	T1	No	Yes	30.514	57077988	7484678
DTcov-0410-212	P145	Patient	Faeces	M	24	Mild	26	T2	No	Yes	28.609	19176613	1346975
DTcov-0410-213	P164	Patient	Sputum	M	50	Moderate	38	T5	Yes	Yes	32.564	53843288	7678037
DTcov-0410-214	P204	Patient	Sputum	F	36	Moderate	65	T1	No	No	35.111	46263064	7579648
DTcov-0410-215	P204	Patient	Pharyngeal	F	36	Moderate	65	T0	No	No	29.475	3288098	787932
DTcov-0410-216	P192	Patient	Pharyngeal	M	19	Mild	9	T0	No	Yes	31.156	27361577	4930202
DTcov-0410-217	P152	Patient	Sputum	M	18	Mild	2	T0	No	Yes	31.925	50415096	4264261
DTcov-0420-218	P204	Patient	Sputum	F	36	Moderate	26	T0	No	No	30.748	55011837	11308397
DTcov-0420-219	P135	Patient	Pharyngeal	F	58	Moderate	9	T0	No	Yes	28.358	48548894	6423551
DTcov-0420-220	P200	Patient	Sputum	M	21	Moderate	8	T0	No	No	29.224	57195874	4573983
DTcov-0420-221	P143	Patient	Sputum	F	40	Moderate	17	T0	No	Yes	26.314	7535894	2716885
DTcov-0420-222	P97	Patient	Sputum	M	42	Moderate	47	T0	No	No	31.013	72064353	15095302
DTcov-0420-223	P98	Patient	Pharyngeal	M	38	Severe	19	T0	No	No	28.179	3548699	1156633
DTcov-0420-224	P283	Patient	Pharyngeal	F	36	Mild	68	T0	No	No	28.788	15380955	2927884
DTcov-0420-225	P110	Patient	Pharyngeal	F	29	Moderate	8	T0	No	Yes	31.824	65911516	4474141
DTcov-0420-226	P171	Patient	Sputum	F	47	Moderate	1	T1	No	Yes	28.276	56589883	7706422
DTcov-0420-227	P190	Patient	Sputum	M	39	Moderate	8	T0	No	Yes	32.154	19560200	2111545

DTcov-0420-228	P155	Patient	Sputum	F	43	Mild	0	T0	No	No	32.004	64306520	5556383
DTcov-0420-229	P147	Patient	Sputum	F	19	Moderate	18	T0	No	No	36.488	62973716	6561775
DTcov-0420-230	P19	Patient	Faeces	F	4	Mild	10	T0	No	No	28.731	64725702	4858865
DTcov-0420-231	P125	Patient	Pharyngeal	M	9	Mild	30	T0	No	No	29.148	57317405	14743450
DTcov-0420-232	P49	Patient	Pharyngeal	M	10	Mild	4	T0	No	No	33.152	4535676	1445844
DTcov-0420-233	P173	Patient	Sputum	M	53	Moderate	20	T0	No	No	34.657	14598246	4078504
DTcov-0420-234	P149	Patient	Pharyngeal	F	9	Moderate	1	T0	No	Yes	33.948	1293834	181154
DTcov-0420-236	P254	Patient	Pharyngeal	M	28	Moderate	11	T0	No	No	28.219	30818135	3135631
DTcov-0420-237	P220	Patient	Faeces	M	27	Moderate	11	T0	No	No	28.435	57402642	2932133
DTcov-0420-238	P105	Patient	Faeces	F	34	Moderate	16	T0	NA	Yes	28.558	68667243	5153931
DTcov-0420-239	P209	Patient	Pharyngeal	F	17	Mild	13	T0	No	No	28.753	60231955	7046836
DTcov-0420-240	P58	Patient	Sputum	F	51	Severe	13	T0	Yes	No	27.142	2637180	1183572
DTcov-0420-241	P57	Patient	Pharyngeal	M	88	Severe	10	T0	NA	No	27.302	3851504	1320663
DTcov-0420-242	P222	Patient	Sputum	M	55	Severe	16	T0	No	No	33.935	59812459	10109730
DTcov-0420-243	P11	Patient	Pharyngeal	F	39	Moderate	7	T0	Yes	No	25.248	60090490	6565897
DTcov-0420-244	P210	Patient	Sputum	F	24	Moderate	2	T0	No	No	31.282	99550056	15047619
DTcov-0420-245	P42	Patient	Sputum	M	57	Moderate	4	T0	No	No	27.342	50703107	7749594
DTcov-0420-246	P43	Patient	Sputum	M	35	Severe	6	T0	Yes	No	32.253	67783680	9753643
DTcov-0420-247	P179	Patient	Sputum	F	22	Moderate	0	T0	No	No	29.632	103653541	12061954
DTcov-0420-248	P257	Patient	Pharyngeal	F	26	Mild	11	T0	No	No	27.486	12432352	2036151
DTcov-0420-249	P36	Patient	Sputum	F	50	Moderate	9	T0	No	No	28.907	20752145	6711526
DTcov-0420-250-sherry-m1	P99	Patient	Sputum	F	56	Moderate	54	T0	No	No	34.615	21419025	3177918
DTcov-0420-251-sherry-m1	P216	Patient	Faeces	M	34	Moderate	4	T0	No	No	29.002	8580244	973096
DTcov-0420-253-sherry-m1	P119	Patient	Faeces	M	37	Moderate	16	T0	No	No	31.077	43003217	1821036
DTcov-0420-254-sherry-m1	P33	Patient	Pharyngeal	F	76	Moderate	14	T0	No	No	28.482	10165659	2332390
DTcov-0420-255-sherry-m1	P27	Patient	Faeces	F	31	Moderate	12	T0	No	No	31.767	41018602	1307989
DTcov-0420-256-sherry-m1	P107	Patient	Faeces	F	0.5	Mild	5	T0	No	No	29.165	2971187	244370
DTcov-0420-257-sherry-m1	P131	Patient	Faeces	F	36	Moderate	34	T0	No	Yes	35.599	9035349	1087618
DTcov-0420-258-sherry-m1	P29	Patient	Pharyngeal	M	80	Severe	24	T0	No	No	26.975	39028109	9890369
DTcov-0420-259-sherry-m1	P73	Patient	Pharyngeal	M	55	Severe	5	T0	No	No	29.656	68823917	10366116
DTcov-0420-260-sherry-m1	P246	Patient	Faeces	M	22	Mild	13	T0	No	No	28.888	38704466	1636579
DTcov-0420-261-sherry-m1	P229	Patient	Sputum	F	20	Mild	5	T0	No	No	38.173	43438133	5871000
DTcov-0420-262-sherry-m1	P160	Patient	Sputum	M	67	Moderate	35	T0	Yes	No	32.256	53649805	6664248
DTcov-0420-263-sherry-m1	P134	Patient	Faeces	M	33	Severe	12	T0	NA	Yes	33.473	53844986	2088559
DTcov-0420-264-sherry-m1	P45	Patient	Sputum	M	50	Severe	6	T0	No	No	33.973	45567728	8085257
DTcov-0420-265-sherry-m1	P46	Patient	Pharyngeal	M	19	Moderate	3	T0	No	No	27.371	38036756	7007809
DTcov-0420-266-sherry-m1	P64	Patient	Pharyngeal	M	69	Severe	17	T0	No	No	29.215	2075266	815025
DTcov-0420-267-sherry-m1	P116	Patient	Faeces	M	32	Moderate	18	T0	No	Yes	28.253	25724168	1704907
DTcov-0420-268-sherry-m1	P50	Patient	Sputum	M	47	Severe	14	T0	NA	Yes	28.693	38753658	6629422
DTcov-0420-269-sherry-m1	P255	Patient	Sputum	F	22	Moderate	4	T0	No	No	34.909	35259162	3149526
DTcov-0420-270-sherry-m1	P10	Patient	Pharyngeal	F	29	Moderate	2	T0	No	No	20.243	5575443	1762476
DTcov-0420-271-sherry-m1	P249	Patient	Pharyngeal	M	29	Moderate	8	T0	Yes	No	27.497	33752361	6718473
DTcov-0420-272-sherry-m1	P206	Patient	Sputum	M	20	Moderate	13	T0	No	No	30.273	68229033	6454160
DTcov-0420-273-sherry-m1	P250	Patient	Sputum	F	6	Moderate	4	T0	No	No	30.547	63799304	9413106
DTcov-0420-274-sherry-m1	P275	Patient	Pharyngeal	F	70	Severe	11	T0	No	No	28.965	34049897	8462865
DTcov-0420-276-sherry-m1	P156	Patient	Sputum	M	42	Mild	0	T0	No	No	29.167	5490595	3141300
DTcov-0420-277-sherry-m1	P232	Patient	Sputum	F	20	Moderate	7	T0	No	No	34.891	8786724	2223628
DTcov-0420-278-sherry-m1	P218	Patient	Pharyngeal	M	37	Moderate	4	T0	No	No	28.487	24788681	5403349
DTcov-0420-279-sherry-m1	P231	Patient	Sputum	F	8	Mild	7	T0	No	No	31.383	91865035	14269445
DTcov-0420-280-sherry-m1	P264	Patient	Sputum	M	16	Mild	8	T0	No	No	28.819	74494097	8537804
DTcov-0420-281-sherry-m1	P236	Patient	Sputum	M	28	Mild	2	T0	No	Yes	32.116	70538046	8590113
DTcov-0420-282-sherry-m1	P213	Patient	Sputum	F	22	Moderate	4	T0	No	No	21.163	68193002	6938703
DTcov-0420-283-sherry-m1	P212	Patient	Sputum	M	21	Moderate	7	T0	No	No	23.497	60378246	7674747
DTcov-0420-284-sherry-m1	P230	Patient	Sputum	M	24	Moderate	4	T0	No	No	26.751	54008656	5774291
DTcov-0420-285-sherry-m1	P240	Patient	Sputum	F	24	Moderate	17	T0	No	No	35.678	47356076	7814848
DTcov-0420-286-sherry-m1	P262	Patient	Sputum	F	40	Moderate	8	T0	No	No	30.017	6408889	1698374
DTcov-0420-287-sherry-m1	P234	Patient	Sputum	M	37	Severe	19	T0	No	Yes	31.834	29188634	3445726
DTcov-0420-288-sherry-m1	P248	Patient	Sputum	F	58	Severe	10	T0	No	No	30.686	59552043	6072345
DTcov-0420-289-sherry-m1	P261	Patient	Sputum	M	22	Moderate	8	T0	No	No	26.312	4512996	1653247
DTcov-0420-290-sherry-m1	P162	Patient	Pharyngeal	M	40	Moderate	13	T0	No	No	22.82	13906631	2324019
DTcov-0420-291-sherry-m1	P245	Patient	Sputum	F	36	Moderate	17	T0	No	No	32.688	50926438	7622354
DTcov-0420-292-sherry-m1	P237	Patient	Faeces	F	23	Mild	8	T0	No	No	28.819	46006479	2692362
DTcov-0420-293-sherry-m1	P242	Patient	Sputum	F	45	Severe	0	T0	No	No	30.191	39366918	6442529
DTcov-0420-295-sherry-m1	P243	Patient	Pharyngeal	M	13	Moderate	0	T0	No	No	28.485	56077015	5714551
DTcov-0420-296-sherry-m1	P215	Patient	Sputum	F	20	Moderate	2	T0	No	Yes	25.039	40367138	3970751
DTcov-0420-297-sherry-m1	P266	Patient	Sputum	M	40	Moderate	11	T0	No	No	32.474	38080775	4702154
DTcov-0420-298-sherry-m1	P268	Patient	Sputum	M	40	Severe	7	T0	No	Yes	28.046	4837720	1526872
DTcov-0420-299	P238	Patient	Sputum	F	18	Mild	8	T0	No	No	23.333	38523982	4612818
DTcov-0420-300	P241	Patient	Pharyngeal	M	39	Moderate	16	T0	Yes	No	28.745	1351504	311139
DTcov-0420-301	P14	Patient	Sputum	M	47	Moderate	50	T0	No	No	33.578	63584065	11968383
DTcov-0420-302	P52	Patient	Pharyngeal	F	2	Moderate	34	T1	No	No	27.761	27406423	3954842
DTcov-0420-303	P31	Patient	Pharyngeal	M	37	Moderate	6	T0	No	No	31.835	72088145	13874159
DTcov-0430-304-sherry-m1	P198	Patient	Sputum	F	21	Moderate	27	T5	Yes	No	26.299	27506133	4811697
DTcov-0430-305-sherry-m1	P156	Patient	Pharyngeal	M	42	Mild	14	T0	No	Yes	31.891	139142783	10233187

DTcov-0430-306-sherry-m1	P172	Patient	Pharyngeal	F	44	Moderate	20	T3	No	No	34.558	60189285	11343374
DTcov-0430-307-sherry-m1	P172	Patient	Sputum	F	44	Moderate	20	T2	No	No	29.785	100680972	14217007
DTcov-0430-308-sherry-m1	P189	Patient	Sputum	F	40	Moderate	16	T1	Yes	No	30.715	97245934	22372884
DTcov-0430-309-sherry-m1	P236	Patient	Sputum	M	28	Mild	16	T1	No	Yes	30.086	107869032	16150753
DTcov-0430-310-sherry-m1	P212	Patient	Pharyngeal	M	21	Moderate	7	T0	No	Yes	30.211	132441427	35965114
DTcov-0430-311-sherry-m1	P212	Patient	Faeces	M	21	Moderate	7	T0	No	Yes	28.549	9004067	5506323
DTcov-0430-312-sherry-m1	P212	Patient	Faeces	M	21	Moderate	21	T1	No	Yes	28.325	105618347	9464302
DTcov-0430-313-sherry-m1	P212	Patient	Sputum	M	21	Moderate	21	T3	No	Yes	31.58	44687628	26060532
DTcov-0430-314-sherry-m1	P212	Patient	Pharyngeal	M	21	Moderate	21	T1	No	Yes	28.819	126366659	23689080
DTcov-0430-315-sherry-m1	P191	Patient	Faeces	F	36	Moderate	8	T0	Yes	Yes	25.467	6359847	3312265
DTcov-0430-316-sherry-m1	P191	Patient	Sputum	F	36	Moderate	8	T0	Yes	Yes	35.02	109372669	11059140
DTcov-0430-317-sherry-m1	P191	Patient	Faeces	F	36	Moderate	22	T1	Yes	No	28.75	103631974	7745885
DTcov-0430-318-sherry-m1	P191	Patient	Sputum	F	36	Moderate	22	T1	Yes	No	29.525	7893568	3598114
DTcov-0430-319-sherry-m1	P60	Patient	Pharyngeal	F	78	Severe	52	T9	Yes	No	26.801	13880423	2786012
DTcov-0430-320-sherry-m1	P195	Patient	Pharyngeal	M	28	Mild	16	T2	Yes	No	28.423	65774703	11641489
DTcov-0430-321-sherry-m1	P196	Patient	Sputum	F	27	Mild	19	T1	Yes	No	33.274	116099897	10911014
DTcov-0430-322-sherry-m1	P162	Patient	Faeces	M	40	Moderate	13	T0	No	No	35.719	11774540	3595293
DTcov-0430-323-sherry-m1	P162	Patient	Pharyngeal	M	40	Moderate	27	T2	No	No	29.156	19016156	3731261
DTcov-0430-324-sherry-m1	P162	Patient	Sputum	M	40	Moderate	27	T2	No	No	26.374	98988646	19012429
DTcov-0430-325-sherry-m1	P162	Patient	Faeces	M	40	Moderate	27	T2	No	No	32.647	98886300	2956373
DTcov-0430-326-sherry-m1	P215	Patient	Faeces	F	20	Moderate	2	T0	No	Yes	29.986	6831626	3128905
DTcov-0430-327-sherry-m1	P215	Patient	Faeces	F	20	Moderate	16	T2	Yes	Yes	30.312	118597372	5698189
DTcov-0430-328-sherry-m1	P215	Patient	Sputum	F	20	Moderate	16	T2	Yes	Yes	29.663	105929555	21012411
DTcov-0430-330-sherry-m1	P164	Patient	Sputum	M	50	Moderate	24	T3	Yes	Yes	29.193	108602520	12985460
DTcov-0430-331-sherry-m1	P161	Patient	Pharyngeal	M	40	Moderate	10	T0	No	Yes	29.198	7435829	2552149
DTcov-0430-332-sherry-m1	P161	Patient	Sputum	M	40	Moderate	27	T2	No	Yes	33.738	4489053	1716715
DTcov-0430-333-sherry-m1	P18	Patient	Sputum	M	74	Severe	57	T0	No	Yes	28.534	59873926	22066609
DTcov-0430-334-sherry-m1	P61	Patient	Sputum	M	82	Severe	34	T0	NA	No	30.2	20392691	3502046
DTcov-0430-335-sherry-m1	P61	Patient	Sputum	M	82	Severe	47	T1	NA	No	30.169	10619694	3685943
DTcov-0430-336-sherry-m	P39	Patient	Sputum	F	64	Moderate	18	T2	Yes	Yes	28.916	68332020	9098303
DTcov-0430-337-sherry-m	P109	Patient	Pharyngeal	F	59	Moderate	49	T0	No	Yes	28.098	77886623	10572684
DTcov-0430-338-sherry-m	P103	Patient	Pharyngeal	M	78	Severe	28	T3	Yes	No	32.87	27531544	2632583
DTcov-0430-339-sherry-m	P239	Patient	Faeces	M	27	Mild	4	T0	No	Yes	26.892	63687856	5173963
DTcov-0430-340-sherry-m	P239	Patient	Faeces	M	27	Mild	14	T1	No	Yes	28.837	84929656	8285147
DTcov-0430-341-sherry-m	P37	Patient	Sputum	F	86	Severe	18	T2	No	Yes	32.877	61637567	53120111
DTcov-0430-342-sherry-m	P44	Patient	Pharyngeal	M	82	Severe	31	T4	No	No	33.802	6422258	2241770
DTcov-0430-343-sherry-m	P48	Patient	Faeces	M	37	Severe	6	T0	Yes	Yes	29.12	6669876	2214770
DTcov-0430-344-sherry-m	P48	Patient	Pharyngeal	M	37	Severe	7	T0	Yes	Yes	29.768	75628309	14554783
DTcov-0430-345-sherry-m	P197	Patient	Faeces	F	28	Mild	4	T0	No	Yes	27.554	13203652	4109667
DTcov-0430-346-sherry-m	P197	Patient	Pharyngeal	F	28	Mild	18	T2	No	No	29.443	34818274	4351352
DTcov-0430-347-sherry-m	P197	Patient	Faeces	F	28	Mild	18	T1	No	No	30.013	73182936	3292477
DTcov-0430-348-sherry-m	P204	Patient	Pharyngeal	F	36	Moderate	67	T1	No	No	29.105	6705892	2299454
DTcov-0430-349-sherry-m	P110	Patient	Sputum	F	29	Moderate	15	T0	No	Yes	28.495	2986645	1651710
DTcov-0430-350-sherry-m	P98	Patient	Pharyngeal	M	38	Severe	19	T1	No	Yes	28.568	5349772	2388661
DTcov-0430-351-sherry-m	P193	Patient	Sputum	M	41	Moderate	20	T2	Yes	Yes	28.829	41952664	8956691
DTcov-0510-352-sherry-m	P168	Patient	Sputum	F	51	Moderate	4	T2	No	Yes	31.025	134090377	17057069
DTcov-0510-353-sherry-m	P168	Patient	Faeces	F	51	Moderate	18	T1	No	Yes	30.733	141049070	5551528
DTcov-0510-354-sherry-m	P102	Patient	Pharyngeal	M	67	Severe	20	T2	No	No	27.852	13232632	3536382
DTcov-0510-355-sherry-m	P102	Patient	Pharyngeal	M	67	Severe	22	T3	No	No	29.324	14851489	3443118
DTcov-0510-356-sherry-m	P102	Patient	Pharyngeal	M	67	Severe	26	T5	No	No	28.315	12730185	3035779
DTcov-0510-357-sherry-m	P60	Patient	Pharyngeal	F	78	Severe	13	T0	Yes	Yes	27.109	7992398	2406103
DTcov-0510-358-sherry-m	P60	Patient	Pharyngeal	F	78	Severe	16	T2	Yes	Yes	27.568	2330418	1115703
DTcov-0510-359-sherry-m	P60	Patient	Pharyngeal	F	78	Severe	22	T5	Yes	Yes	28.139	6964398	3022174
DTcov-0510-360-sherry-m	P60	Patient	Pharyngeal	F	78	Severe	46	T7	Yes	No	27.699	8814253	3279839
DTcov-0510-361-sherry-m	P60	Patient	Pharyngeal	F	78	Severe	49	T8	Yes	No	28.575	18658603	3462807
DTcov-0510-362-sherry-m	P165	Patient	Sputum	M	33	Moderate	6	T1	No	Yes	31.633	131679157	15714915
DTcov-0510-363-sherry-m	P37	Patient	Sputum	F	86	Severe	7	T0	No	Yes	15.974	15428081	3067003
DTcov-0510-364-sherry-m	P37	Patient	Pharyngeal	F	86	Severe	8	T1	No	Yes	28.27	6598953	2766685
DTcov-0510-365-sherry-m	P37	Patient	Sputum	F	86	Severe	8	T1	No	Yes	28.645	3952892	2749567
DTcov-0510-366-sherry-m	P37	Patient	Pharyngeal	F	86	Severe	11	T2	No	Yes	28.713	8389835	5522239
DTcov-0510-367-sherry-m	P37	Patient	Pharyngeal	F	86	Severe	16	T4	No	Yes	28.462	90938665	78558065
DTcov-0511-368-sherry-m	P37	Patient	Pharyngeal	F	86	Severe	12	T3	No	Yes	23.893	3804771	674686
DTcov-0511-369-sherry-m	P37	Patient	Sputum	F	86	Severe	23	T3	No	Yes	27.075	6570723	1595614
DTcov-0511-370-sherry-m	P30	Patient	Pharyngeal	M	73	Moderate	14	T1	No	No	26.784	35708927	5799395
DTcov-0511-371-sherry-m	P30	Patient	Pharyngeal	M	73	Moderate	43	T2	No	Yes	28.699	4768988	2252819
DTcov-0511-372-sherry-m	P165	Patient	Sputum	M	33	Moderate	6	T2	No	Yes	26.951	29975007	7041857
DTcov-0511-373-sherry-m	P165	Patient	Pharyngeal	M	33	Moderate	37	T0	No	No	28.79	15148627	4399092
DTcov-0511-374-sherry-m	P165	Patient	Pharyngeal	M	33	Moderate	55	T1	No	No	28.773	9052492	3057907
DTcov-0511-375-sherry-m	P102	Patient	Pharyngeal	M	67	Severe	15	T0	No	No	28.706	10149556	3630970
DTcov-0511-376-sherry-m	P102	Patient	Pharyngeal	M	67	Severe	25	T4	No	No	27.103	13437103	5013906
DTcov-0511-377-sherry-m	P168	Patient	Sputum	F	51	Moderate	1	T1	No	Yes	22.859	1826840	625738
DTcov-0511-378-sherry-m	P60	Patient	Pharyngeal	F	78	Severe	19	T4	Yes	Yes	28.336	15190299	6175012
DTcov-0511-379-sherry-m	P60	Patient	Pharyngeal	F	78	Severe	24	T6	Yes	Yes	28.038	12014765	3840404
DTcov-0522-380-sherry-m1	P249	Patient	Sputum	M	29	Moderate	44	T0	Yes	No	31.943	44495706	5090310

DTcov-0522-381-sherry-m1	P244	Patient	Faeces	M	26	Moderate	27	T0	No	No	31.461	45231315	3278704
DTcov-0522-382-sherry-m1	P232	Patient	Sputum	F	20	Moderate	41	T1	No	Yes	31.933	41668716	5230357
DTcov-0522-383-sherry-m1	P214	Patient	Pharyngeal	F	52	Moderate	22	T0	No	Yes	28.95	1465945	375634
DTcov-0522-384-sherry-m1	P210	Patient	Faeces	F	24	Moderate	22	T0	No	Yes	30.142	35509271	3090556
DTcov-0522-385-sherry-m1	P236	Patient	Faeces	M	28	Mild	22	T0	No	Yes	31.184	38608933	2089758
DTcov-0522-386-sherry-m1	P230	Patient	Sputum	M	24	Moderate	40	T2	No	No	30.291	11957314	3258419
DTcov-0522-387-sherry-m1	P228	Patient	Sputum	F	22	Mild	23	T1	No	No	30.162	44630062	7055497
DTcov-0522-388-sherry-m1	P252	Patient	Sputum	F	28	Mild	41	T1	No	No	33.608	44759042	4863332
DTcov-0522-389-sherry-m1	P262	Patient	Pharyngeal	F	40	Moderate	26	T0	No	No	28.775	1602083	406442
DTcov-0522-390-sherry-m1	P169	Patient	Faeces	M	17	Moderate	29	T2	No	Yes	24.745	42494048	5646392
DTcov-0522-391-sherry-m1	P196	Patient	Sputum	F	27	Mild	45	T2	Yes	No	32.711	44182699	4553839
DTcov-0522-392-sherry-m1	P245	Patient	Sputum	F	36	Moderate	43	T2	Yes	No	32.535	47965880	6957860
DTcov-0522-393-sherry-m1	P245	Patient	Sputum	F	36	Moderate	25	T1	No	Yes	31.416	50263232	11190682
DTcov-0522-394-sherry-m1	P237	Patient	Sputum	F	23	Mild	46	T1	Yes	No	29.768	876146	187432
DTcov-0522-395-sherry-m1	P246	Patient	Sputum	M	22	Mild	29	T0	No	No	31.846	4982227	1159505
DTcov-0522-396-sherry-m1	P239	Patient	Faeces	M	27	Mild	22	T2	No	Yes	27.168	70855990	6980320
DTcov-0522-397-sherry-m1	P266	Patient	Pharyngeal	M	40	Moderate	39	T0	No	No	28.325	5263448	729770
DTcov-0529-398-sherry-m1	P47	Patient	Pharyngeal	M	0.9	Moderate	6	T0	Yes	No	31.915	35623632	6351146
DTcov-0529-399-sherry-m1	P47	Patient	Faeces	M	0.9	Moderate	6	T0	Yes	No	28.993	54173161	4346805
DTcov-0529-400-sherry-m1	P47	Patient	Faeces	M	0.9	Moderate	24	T2	Yes	No	27.671	66163733	1816887
DTcov-0529-401-sherry-m1	P172	Patient	Pharyngeal	F	44	Moderate	14	T1	No	Yes	31.252	56116682	16533768
DTcov-0529-402-sherry-m1	P172	Patient	Sputum	F	44	Moderate	14	T0	No	Yes	30.185	56502158	5960677
DTcov-0529-403-sherry-m1	P172	Patient	Sputum	F	44	Moderate	18	T1	No	No	28.22	47444175	4098486
DTcov-0529-404-sherry-m1	P172	Patient	Pharyngeal	F	44	Moderate	18	T2	No	No	28.952	35265685	3602697
DTcov-0529-405-sherry-m1	P212	Patient	Sputum	M	21	Moderate	11	T1	No	Yes	16.829	56612231	8346704
DTcov-0529-406-sherry-m1	P212	Patient	Sputum	M	21	Moderate	19	T2	No	Yes	30.825	58903804	4885139
DTcov-0529-407-sherry-m1	P62	Patient	Sputum	M	52	Moderate	15	T1	Yes	Yes	28.482	689989	232958
DTcov-0529-408-sherry-m1	P62	Patient	Sputum	M	52	Moderate	28	T2	Yes	Yes	28.284	1704560	600291
DTcov-0529-409-sherry-m1	P56	Patient	Pharyngeal	M	59	Moderate	75	T1	No	No	28.974	2042967	435590
DTcov-0529-410-sherry-m1	P56	Patient	Sputum	M	59	Moderate	75	T2	No	No	30.69	56906224	12502831
DTcov-0529-411-sherry-m1	P56	Patient	Faeces	M	59	Moderate	75	T1	No	No	29.504	53215574	3741412
DTcov-0529-412-sherry-m1	P215	Patient	Pharyngeal	F	20	Moderate	3	T0	No	Yes	29.862	47444141	19322092
DTcov-0529-413-sherry-m1	P215	Patient	Faeces	F	20	Moderate	7	T1	Yes	Yes	26.327	42891152	3057885
DTcov-0529-414-sherry-m1	P215	Patient	Sputum	F	20	Moderate	7	T1	Yes	Yes	30.63	5679339	665695
DTcov-0529-416-sherry-m1	P215	Patient	Pharyngeal	F	20	Moderate	13	T1	Yes	Yes	28.847	30270633	5737512
DTcov-0529-417-sherry-m1	P69	Patient	Pharyngeal	F	84	Severe	32	T2	No	No	28.209	5982987	519185
DTcov-0529-418-sherry-m1	P69	Patient	Pharyngeal	F	84	Severe	37	T3	No	No	28.377	35525371	1589805
DTcov-0529-419-sherry-m1	P28	Patient	Faeces	F	33	Moderate	18	T0	Yes	Yes	34.952	46843399	1852168
DTcov-0529-420-sherry-m1	P28	Patient	Sputum	F	33	Moderate	19	T2	Yes	Yes	31.382	57045536	6763026
DTcov-0529-422-sherry-m1	P47	Patient	Faeces	M	0.9	Moderate	10	T1	Yes	No	28.445	7642354	771815
DTcov-0529-423-sherry-m1	P27	Patient	Faeces	F	31	Moderate	12	T1	No	No	35.87	4408768	527840
DTcov-0529-424-sherry-m1	P276	Patient	Sputum	F	66	Severe	2	T0	No	No	23.464	1723831	349731
DTcov-0529-425-sherry-m1	P276	Patient	Pharyngeal	F	66	Severe	26	T0	No	No	28.499	4986763	682240
DTcov-0529-426-sherry-m1	P277	Patient	Pharyngeal	M	67	Severe	22	T1	No	No	28.5	2202638	295940
DTcov-0529-428-sherry-m1	P277	Patient	Faeces	M	67	Severe	6	T0	No	No	28.579	2023107	370037
DTcov-0529-429-sherry-m1	P279	Patient	Sputum	F	44	Moderate	16	T0	No	No	27.117	42053349	4551537
DTcov-0529-430-sherry-m1	P280	Patient	Sputum	M	75	Severe	14	T0	No	No	26.777	3177133	950783
DTcov-0529-431-sherry-m1	P281	Patient	Sputum	M	10	Mild	14	T0	No	No	28.42	27423928	3083860
DTcov-0529-432-sherry-m1	P282	Patient	Sputum	M	20	Severe	18	T0	No	No	30.15	38512920	5630151
DTcov-0603-433-sherry-m1	P230	Patient	Pharyngeal	M	24	Moderate	6	T0	No	No	26.491	24983022	1367016
DTcov-0603-434-sherry-m1	P230	Patient	Faeces	M	24	Moderate	6	T0	No	No	26.784	30229104	3647190
DTcov-0603-435-sherry-m1	P230	Patient	Sputum	M	24	Moderate	14	T1	No	No	28.401	18720865	2469759
DTcov-0603-436-sherry-m1	P230	Patient	Pharyngeal	M	24	Moderate	14	T1	No	No	26.947	23331172	1628015
DTcov-0603-437-sherry-m1	P230	Patient	Faeces	M	24	Moderate	16	T1	No	No	27.883	53029021	3551402
DTcov-0603-438-sherry-m1	P60	Patient	Faeces	F	78	Severe	16	T0	Yes	Yes	27.602	2932008	743582
DTcov-0603-439-sherry-m1	P20	Patient	Sputum	F	63	Moderate	17	T1	No	Yes	28.813	43848273	6552377
DTcov-0603-440-sherry-m1	P99	Patient	Pharyngeal	F	56	Moderate	54	T0	No	No	28.971	48654292	9851414
DTcov-0603-441-sherry-m1	P83	Patient	Sputum	M	63	Moderate	15	T1	No	Yes	30.257	42090491	4046649
DTcov-0603-442-sherry-m1	P83	Patient	Sputum	M	63	Moderate	22	T2	No	Yes	27.719	37665821	3894073
DTcov-0603-444-sherry-m1	P51	Patient	Pharyngeal	F	72	Severe	21	T1	NA	Yes	25.437	7301335	669955
DTcov-0603-445-sherry-m1	P51	Patient	Sputum	F	72	Severe	28	T1	NA	Yes	27.342	2633782	381291
DTcov-0603-446-sherry-m1	P173	Patient	Faeces	M	53	Moderate	24	T0	No	Yes	31.157	35807624	1476574
DTcov-0603-447-sherry-m1	P13	Patient	Sputum	M	33	Moderate	62	T3	No	No	28.182	29708719	4925196
DTcov-0603-448-sherry-m1	P43	Patient	Sputum	M	35	Severe	20	T2	Yes	Yes	29.468	36695981	3103985
DTcov-0603-449-sherry-m1	P5	Patient	Sputum	M	49	Severe	19	T1	Yes	Yes	31.475	43773673	5749903
DTcov-0603-450-sherry-m1	P21	Patient	Pharyngeal	F	62	Severe	15	T1	No	No	29.745	45010725	7787269
DTcov-0603-451-sherry-m1	P44	Patient	Pharyngeal	M	82	Severe	25	T3	No	No	25.552	1917108	342462
DTcov-0603-452-sherry-m1	P174	Patient	Sputum	M	30	Moderate	11	T1	No	Yes	29.002	20051469	3312012
DTcov-0603-453-sherry-m1	P121	Patient	Sputum	F	66	Moderate	15	T0	Yes	Yes	28.45	35058125	3848455
DTcov-0603-456-sherry-m1	P195	Patient	Sputum	M	28	Mild	12	T0	Yes	Yes	27.687	925798	133960
DTcov-0603-457-sherry-m1	P195	Patient	Pharyngeal	M	28	Mild	12	T1	Yes	Yes	29.961	37307653	13187721
DTcov-0603-458-sherry-m1	P195	Patient	Sputum	M	28	Mild	20	T1	Yes	No	34.687	30706008	4606875
DTcov-0603-459-sherry-m1	P195	Patient	Pharyngeal	M	28	Mild	20	T3	Yes	No	29.448	33640123	9643000
DTcov-0603-460-sherry-m1	P195	Patient	Sputum	M	28	Mild	44	T2	Yes	No	27.624	36573810	3632132

DTcov-0603-461-sherry-m1	P135	Patient	Faeces	F	58	Moderate	13	T1	No	Yes	32.004	34661043	1200550
DTcov-0603-462-sherry-m1	P103	Patient	Sputum	M	78	Severe	8	T0	Yes	Yes	26.89	10163968	2264457
DTcov-0603-463-sherry-m1	P103	Patient	Pharyngeal	M	78	Severe	27	T2	Yes	No	29.25	9770802	1177144
DTcov-0603-464-sherry-m1	P103	Patient	Sputum	M	78	Severe	27	T2	Yes	No	28.729	22455464	1841975
DTcov-0603-465-sherry-m1	P103	Patient	Sputum	M	78	Severe	19	T1	Yes	No	29.034	16034147	1657346
DTcov-0603-466-sherry-m1	P103	Patient	Pharyngeal	M	78	Severe	19	T1	Yes	No	24.949	2856790	584188
DTcov-0603-467-sherry-m1	P103	Patient	Pharyngeal	M	78	Severe	35	T5	Yes	No	27.196	1812627	475393
DTcov-0603-468-sherry-m1	P107	Patient	Faeces	F	0.5	Mild	14	T1	No	No	27.367	36965508	1484193
DTcov-0603-469-sherry-m1	P68	Patient	Pharyngeal	M	57	Severe	53	T2	NA	No	25.774	3764651	683492
DTcov-0603-470-sherry-m1	P125	Patient	Faeces	M	9	Mild	64	T1	No	No	29.776	49723502	2335608
DTcov-0603-471-sherry-m1	P125	Patient	Pharyngeal	M	9	Mild	64	T1	No	No	30.546	45096289	10931822
DTcov-0603-472-sherry-m1	P288	Patient	Sputum	F	40	Mild	42	T0	No	No	34.641	40964346	5518967
DTcov-0603-473-sherry-m1	P265	Patient	Pharyngeal	F	23	Mild	18	T0	No	No	25.937	1222056	448968
DTcov-0603-474-sherry-m1	P265	Patient	Sputum	F	23	Mild	18	T0	No	No	26.969	22226749	3613141
DTcov-0603-475-sherry-m1	P164	Patient	Faeces	M	50	Moderate	6	T0	No	Yes	29.816	42724103	1642559
DTcov-0603-476-sherry-m1	P164	Patient	Sputum	M	50	Moderate	6	T1	No	Yes	31.384	40880987	2768018
DTcov-0603-477-sherry-m1	P164	Patient	Sputum	M	50	Moderate	36	T4	Yes	Yes	30.766	30416693	2812306
DTcov-0603-478-sherry-m1	P164	Patient	Faeces	M	50	Moderate	46	T1	Yes	Yes	28.671	34756362	3454135
DTcov-0603-479-sherry-m1	P65	Patient	Faeces	M	6	Mild	13	T1	No	No	27.642	43893979	2381605
DTcov-0603-480-sherry-m1	P269	Patient	Sputum	M	21	Moderate	1	T0	No	Yes	29.393	40061279	5589195
DTcov-0603-481-sherry-m1	P169	Patient	Faeces	M	17	Moderate	3	T0	No	Yes	28.924	40698976	1937710
DTcov-0603-482-sherry-m1	P169	Patient	Sputum	M	17	Moderate	3	T0	No	Yes	20.941	6432491	411424
DTcov-0603-483-sherry-m1	P169	Patient	Pharyngeal	M	17	Moderate	13	T1	No	Yes	29.938	35077363	5480409
DTcov-0603-484-sherry-m1	P169	Patient	Sputum	M	17	Moderate	13	T1	No	Yes	29.832	35283690	3309893
DTcov-0603-485-sherry-m1	P169	Patient	Pharyngeal	M	17	Moderate	21	T2	No	Yes	28.776	10951457	1881395
DTcov-0603-486-sherry-m1	P169	Patient	Sputum	M	17	Moderate	21	T2	No	Yes	32.182	9191584	718640
DTcov-0603-487-sherry-m1	P169	Patient	Faeces	M	17	Moderate	21	T1	No	Yes	30.896	41847878	1373268
DTcov-0604-488-sherry-m1	P175	Patient	Faeces	M	58	Moderate	0	T0	No	Yes	33.446	87126690	2632471
DTcov-0604-489-sherry-m1	P166	Patient	Sputum	M	51	Moderate	6	T1	No	Yes	33.076	102899710	12546707
DTcov-0604-490-sherry-m1	P167	Patient	Sputum	F	40	Moderate	11	T1	No	Yes	28.707	109478106	18504724
DTcov-0604-491-sherry-m1	P135	Patient	Faeces	F	58	Moderate	21	T2	No	Yes	32.518	105886296	1786000
DTcov-0604-492-sherry-m1	P144	Patient	Sputum	M	35	Moderate	14	T0	No	Yes	34.748	117012460	4056508
DTcov-0604-493-sherry-m1	P161	Patient	Sputum	M	40	Moderate	23	T1	No	Yes	30.64	40270262	2547245
DTcov-0604-494-sherry-m1	P183	Patient	Faeces	M	49	Moderate	9	T0	No	Yes	29.084	28114461	2109187
DTcov-0604-495-sherry-m1	P183	Patient	Faeces	M	49	Moderate	24	T1	No	Yes	31.07	25005208	1131468
DTcov-0604-496-sherry-m1	P253	Patient	Pharyngeal	M	4	Mild	30	T1	No	Yes	30.794	5504733	1219467
DTcov-0604-497-sherry-m1	P198	Patient	Pharyngeal	F	21	Moderate	13	T0	Yes	No	29.975	3796027	1675294
DTcov-0604-498-sherry-m1	P198	Patient	Sputum	F	21	Moderate	13	T1	Yes	No	28.348	46059154	4940110
DTcov-0604-499-sherry-m1	P198	Patient	Pharyngeal	F	21	Moderate	15	T1	Yes	No	30.381	12025448	2591336
DTcov-0604-500-sherry-m1	P189	Patient	Faeces	F	40	Moderate	27	T1	Yes	No	31.079	13726450	1001847
DTcov-0604-501-sherry-m1	P198	Patient	Pharyngeal	F	21	Moderate	21	T2	Yes	No	28.644	9097351	516996
DTcov-0604-502-sherry-m1	P198	Patient	Sputum	F	21	Moderate	21	T2	Yes	No	29.532	8504562	1057460
DTcov-0604-503-sherry-m1	P198	Patient	Sputum	F	21	Moderate	23	T3	Yes	No	31.402	33783899	6721435
DTcov-0604-504-sherry-m1	P198	Patient	Sputum	F	21	Moderate	25	T4	Yes	No	31.206	42925734	5084257
DTcov-0604-505-sherry-m1	P199	Patient	Pharyngeal	F	32	Mild	7	T0	No	Yes	22.947	4403345	879323
DTcov-0604-506-sherry-m1	P199	Patient	Pharyngeal	F	32	Mild	23	T1	No	Yes	29.243	25957171	3490953
DTcov-0604-507-sherry-m1	P199	Patient	Faeces	F	32	Mild	31	T1	No	Yes	31.335	44819115	2491011
DTcov-0604-508-sherry-m1	P201	Patient	Pharyngeal	F	76	Severe	5	T0	NA	No	30.208	2230478	434548
DTcov-0604-509-sherry-m1	P247	Patient	Faeces	F	23	Mild	3	T0	No	No	27.136	24015815	1057915
DTcov-0604-510-sherry-m1	P247	Patient	Faeces	F	23	Mild	21	T1	No	No	29.115	29828809	3593237
DTcov-0604-511-sherry-m1	P162	Patient	Pharyngeal	M	40	Moderate	23	T1	No	No	31.493	47906892	4452146
DTcov-0604-512-sherry-m1	P162	Patient	Faeces	M	40	Moderate	23	T1	No	No	30.776	46446067	1897332
DTcov-0604-513-sherry-m1	P162	Patient	Sputum	M	40	Moderate	23	T1	No	No	31.652	41101503	5091451
DTcov-0604-514-sherry-m1	P162	Patient	Faeces	M	40	Moderate	29	T3	No	No	30.463	38130645	1332947
DTcov-0604-515-sherry-m1	P237	Patient	Sputum	F	23	Mild	44	T0	Yes	No	30.853	49417628	5037866
DTcov-0604-516-sherry-m1	P197	Patient	Pharyngeal	F	28	Mild	14	T1	No	Yes	32.904	50396661	15598721
DTcov-0604-517-sherry-m1	P197	Patient	Faeces	F	28	Mild	28	T2	No	No	30.52	806552	113974
DTcov-0604-518-sherry-m1	P193	Patient	Pharyngeal	M	41	Moderate	2	T0	No	Yes	22.731	4080724	384963
DTcov-0604-519-sherry-m1	P193	Patient	Sputum	M	41	Moderate	14	T1	Yes	Yes	31.138	54307166	6466620
DTcov-0604-520-sherry-m1	P193	Patient	Pharyngeal	M	41	Moderate	16	T1	Yes	Yes	29.998	773651	164040
DTcov-0604-521-sherry-m1	P156	Patient	Faeces	M	42	Mild	0	T0	No	No	29.189	22298847	1276468
DTcov-0604-522-sherry-m1	P156	Patient	Faeces	M	42	Mild	14	T1	No	Yes	32.784	41862344	2583027
DTcov-0604-523-sherry-m1	P214	Patient	Sputum	F	52	Moderate	24	T1	No	Yes	29.22	24098185	6966102
DTcov-0604-524-sherry-m1	P189	Patient	Pharyngeal	F	40	Moderate	7	T1	Yes	Yes	27.505	25546158	8247656
DTcov-0604-525-sherry-m1	P189	Patient	Sputum	F	40	Moderate	7	T0	Yes	Yes	29.084	54946771	4517099
DTcov-0604-526-sherry-m1	P189	Patient	Faeces	F	40	Moderate	7	T0	Yes	Yes	27.934	7365860	1001688
Y2	P113	Patient	Sputum	M	57	Moderate	10	T0	No	Yes	20.15	2905953	205752
Y3	P122	Patient	Sputum	F	45	Moderate	15	T0	No	Yes	25.387	14759523	736811
Y4	P116	Patient	Sputum	M	32	Moderate	19	T0	No	Yes	27.859	11334827	989139
Y5	P114	Patient	Sputum	M	36	Moderate	13	T1	No	Yes	29.194	4349585	811926
DTcov-0403-Y7	P137	Patient	Pharyngeal	M	30	Mild	8	T1	No	Yes	28.512	640377	221920
DTcov-0403-Y9	P137	Patient	Sputum	M	30	Mild	9	T2	No	Yes	31.341	554802	200108
DTcov-0427-Y15-sherry-m1	P77	Patient	Sputum	F	33	Moderate	24	T0	No	No	28.482	62633725	5146146
DTcov-0427-Y16-sherry-m1	P91	Patient	Pharyngeal	F	37	Moderate	17	T0	No	No	26.298	1690991	1147483

DTcov-0427-Y17	P17	Patient	Pharyngeal	F	72	Severe	21	T1	No	No	25.25	14837039	1992908
DTcov-0427-Y18-sherry-m1	P18	Patient	Pharyngeal	M	74	Severe	25	T3	No	No	29.671	464038	300876
DTcov-0427-Y19-sherry-m1	P157	Patient	Sputum	M	23	Mild	9	T0	Yes	No	30.111	782976	521608
DTcov-0427-Y20	P17	Patient	Sputum	F	72	Severe	63	T3	No	No	27.913	3640841	2777103
DTcov-0427-Y21-sherry-m1	P18	Patient	Sputum	M	74	Severe	70	T1	No	No	29.21	56955168	5014298
DTcov-NC-B1-sherry-m1	H1	Healthy	Faeces	M	40	Healthy	0	T0	No	No	40	11103500	274121
DTcov-NC-B2-sherry-m1	H2	Healthy	Faeces	F	33	Healthy	0	T0	No	No	33.5	14887846	494231
DTcov-NC-B3-sherry-m1	H3	Healthy	Faeces	M	36	Healthy	0	T0	No	No	32.746	12370262	1395918
DTcov-NC-B4-sherry-m1	H4	Healthy	Faeces	F	32	Healthy	0	T0	No	No	33.481	7185766	243923
DTcov-NC-B5-sherry-m1	H5	Healthy	Faeces	F	32	Healthy	0	T0	No	No	40	8951127	1204783
DTcov-NC-B6-sherry-m1	H6	Healthy	Faeces	M	32	Healthy	0	T0	No	No	33.662	14714003	347233
DTcov-NC-B7-sherry-m1	H7	Healthy	Faeces	M	39	Healthy	0	T0	No	No	33.313	14606855	291237
DTcov-NC-B8-sherry-m1	H8	Healthy	Faeces	M	51	Healthy	0	T0	No	No	34.808	12120862	259671
DTcov-NC-T1-sherry-m1	H1	Healthy	Sputum	M	40	Healthy	0	T0	No	No	35.946	1514998	638736
DTcov-NC-T2-sherry-m1	H2	Healthy	Sputum	F	33	Healthy	0	T0	No	No	30.891	15165093	1565794
DTcov-NC-T3-sherry-m1	H3	Healthy	Sputum	M	36	Healthy	0	T0	No	No	31.76	22247222	1649872
DTcov-NC-T4-sherry-m1	H4	Healthy	Sputum	F	32	Healthy	0	T0	No	No	32.559	4452642	765516
DTcov-NC-T5-sherry-m1	H5	Healthy	Sputum	F	32	Healthy	0	T0	No	No	31.477	4672619	894809
DTcov-NC-T6-sherry-m1	H6	Healthy	Sputum	M	32	Healthy	0	T0	No	No	37.667	17313418	1679014
DTcov-NC-T7-sherry-m1	H7	Healthy	Sputum	M	39	Healthy	0	T0	No	No	34.526	40285851	2798816
DTcov-NC-T8-sherry-m1	H8	Healthy	Sputum	M	51	Healthy	0	T0	No	No	33.082	6101879	1077251
DTcov-NC-Y1-sherry-m1	H1	Healthy	Pharyngeal	M	40	Healthy	0	T0	No	No	33.379	10524232	2661398
DTcov-NC-Y2-sherry-m1	H2	Healthy	Pharyngeal	F	33	Healthy	0	T0	No	No	31.052	3394647	2330412
DTcov-NC-Y3-sherry-m1	H3	Healthy	Pharyngeal	M	36	Healthy	0	T0	No	No	40	5345972	2161861
DTcov-NC-Y4-sherry-m1	H4	Healthy	Pharyngeal	F	32	Healthy	0	T0	No	No	31.729	26071758	4137917
DTcov-NC-Y5-sherry-m1	H5	Healthy	Pharyngeal	F	32	Healthy	0	T0	No	No	31.466	9107424	2511179
DTcov-NC-Y6-sherry-m1	H6	Healthy	Pharyngeal	M	32	Healthy	0	T0	No	No	40	14781054	2830752
DTcov-NC-Y7-sherry-m1	H7	Healthy	Pharyngeal	M	39	Healthy	0	T0	No	No	33.994	5591165	2242409
DTcov-NC-Y8-sherry-m1	H8	Healthy	Pharyngeal	M	51	Healthy	0	T0	No	No	34.803	33385818	3485836
Dtcov-1B-m	H14	Healthy	Faeces	F	4	Healthy	0	T0	No	No	27.161	7633616	4896129
Dtcov-1Y-m	H14	Healthy	Pharyngeal	F	4	Healthy	0	T0	No	No	34.842	8651039	1506545
Dtcov-2B-m	H16	Healthy	Faeces	M	7	Healthy	0	T0	No	No	29.997	1599283	796295
Dtcov-2Y-m	H16	Healthy	Pharyngeal	M	7	Healthy	0	T0	No	No	31.779	1884787	934676
Dtcov-3B-m	H22	Healthy	Faeces	F	34	Healthy	31	T1	No	No	30.287	19139993	3027091
Dtcov-3Y-m	H22	Healthy	Pharyngeal	F	34	Healthy	30	T1	No	No	28.664	3119111	854332
Dtcov-4B-m	H4	Healthy	Faeces	F	32	Healthy	102	T1	No	No	28.569	39327859	1987472
Dtcov-6B-m	H11	Healthy	Faeces	M	3.5	Healthy	0	T0	No	No	28.698	28909253	2328531
Dtcov-7B-m	H19	Healthy	Faeces	F	24	Healthy	0	T0	No	No	28.832	34545048	1229299
Dtcov-7T-m	H19	Healthy	Sputum	F	24	Healthy	0	T0	No	No	31.056	21891084	3018499
Dtcov-7Y-m	H19	Healthy	Pharyngeal	F	24	Healthy	0	T0	No	No	31.043	2324607	828846
Dtcov-8B-m	H8	Healthy	Faeces	M	51	Healthy	88	T1	No	No	28.296	6606577	4290909
Dtcov-8Y-m	H8	Healthy	Pharyngeal	M	51	Healthy	86	T1	No	No	NA	1290086	647573
Dtcov-9B-m	H32	Healthy	Faeces	M	78	Healthy	0	T0	No	No	30.868	17159092	936876
Dtcov-9Y-m	H32	Healthy	Pharyngeal	M	78	Healthy	0	T0	No	No	NA	1568942	798529
Dtcov-10B-m	H31	Healthy	Faeces	F	77	Healthy	0	T0	No	No	35.962	6420103	962461
Dtcov-10Y-m	H31	Healthy	Pharyngeal	F	77	Healthy	0	T0	No	No	29.907	5968276	1098311
Dtcov-11B-m	H20	Healthy	Faeces	F	24	Healthy	0	T0	No	No	36.172	39483274	1395204
Dtcov-11T-m	H20	Healthy	Sputum	F	24	Healthy	0	T0	No	No	35.216	10053664	1125345
Dtcov-11Y-m	H20	Healthy	Pharyngeal	F	24	Healthy	0	T0	No	No	31.666	1757447	826699
Dtcov-12Y-m	H23	Healthy	Pharyngeal	F	37	Healthy	0	T0	No	No	NA	1802441	816317
Dtcov-13B-m	H22	Healthy	Faeces	F	34	Healthy	0	T0	No	No	33.726	4245248	2241981
Dtcov-13Y-m	H22	Healthy	Pharyngeal	F	34	Healthy	0	T0	No	No	32.224	940056	591811
Dtcov-14Y-m	H28	Healthy	Pharyngeal	F	61	Healthy	0	T0	No	No	34.048	11992812	1778355
Dtcov-15B-m	H5	Healthy	Faeces	F	32	Healthy	86	T1	No	No	NA	1939097	650569
Dtcov-16Y-m	H4	Healthy	Pharyngeal	F	32	Healthy	86	T1	No	No	36.182	1513932	612709
Dtcov-17B-m	H1	Healthy	Faeces	M	40	Healthy	100	T1	No	No	27.845	5513146	868048
Dtcov-17Y-m	H1	Healthy	Pharyngeal	M	40	Healthy	86	T1	No	No	33.29	3711749	924353
Dtcov-18Y-m	H28	Healthy	Pharyngeal	F	61	Healthy	30	T1	No	No	30.606	1891041	769746
Dtcov-19B-m	H7	Healthy	Faeces	M	39	Healthy	86	T1	No	No	26.962	7738306	1420512
Dtcov-19Y-m	H7	Healthy	Pharyngeal	M	39	Healthy	90	T1	No	No	31.905	4024579	1309939
Dtcov-20Y-m	H24	Healthy	Pharyngeal	F	38	Healthy	0	T0	No	No	NA	1993172	834031
Dtcov-21Y-m	H3	Healthy	Pharyngeal	M	36	Healthy	86	T1	No	No	25.894	1133287	658259
Dtcov-22B-m	H2	Healthy	Faeces	F	33	Healthy	92	T1	No	No	NA	5606106	884662
Dtcov-23B-m	H9	Healthy	Faeces	M	2	Healthy	0	T0	No	No	28.908	33023219	1186575
Dtcov-24B-m	H10	Healthy	Faeces	M	2	Healthy	0	T0	No	No	28.056	11643386	1161688
Dtcov-24Y-m	H10	Healthy	Pharyngeal	M	2	Healthy	0	T0	No	No	30.929	5664279	912448
Dtcov-25B-m	H18	Healthy	Faeces	F	23	Healthy	20	T1	No	No	28.353	35397865	1149193
Dtcov-25T-m	H18	Healthy	Sputum	F	23	Healthy	19	T1	No	No	NA	28812948	2805149
Dtcov-25Y-m	H18	Healthy	Pharyngeal	F	23	Healthy	20	T0	No	No	NA	1606600	858178
Dtcov-26B-m	H20	Healthy	Faeces	F	24	Healthy	20	T1	No	No	27.46	39014191	2060812
Dtcov-27B-m	H19	Healthy	Faeces	F	24	Healthy	20	T1	No	No	31.386	41961263	4271793
Dtcov-27T-m	H19	Healthy	Sputum	F	24	Healthy	19	T1	No	No	NA	22461646	4342321
Dtcov-27Y-m	H19	Healthy	Pharyngeal	F	24	Healthy	19	T1	No	No	30.844	19923957	2408961
Dtcov-28B-m	H15	Healthy	Faeces	F	6	Healthy	0	T0	No	No	35.168	4679073	3684947

Dtcov-28Y-m	H15	Healthy	Pharyngeal	F	6	Healthy	0	T0	No	No	31.162	28829238	2472469
Dtcov-29B-m	H29	Healthy	Faeces	M	63	Healthy	0	T0	No	No	26.395	42100338	4886507
Dtcov-29Y-m	H29	Healthy	Pharyngeal	M	63	Healthy	0	T0	No	No	NA	42884479	3475940
Dtcov-30B-m	H12	Healthy	Faeces	M	4	Healthy	0	T0	No	No	30.974	34264708	3079092
Dtcov-30Y-m	H12	Healthy	Pharyngeal	M	4	Healthy	0	T0	No	No	30.119	20535350	2099653
Dtcov-31B-m	H27	Healthy	Faeces	F	60	Healthy	0	T0	No	No	27.937	8966108	1731179
Dtcov-31Y-m	H27	Healthy	Pharyngeal	F	60	Healthy	0	T0	No	No	33.663	36716624	2817404
Dtcov-32B-m	H21	Healthy	Faeces	F	26	Healthy	0	T0	No	No	33.107	26279281	2274480
Dtcov-32T-m	H21	Healthy	Sputum	F	26	Healthy	0	T0	No	No	34.938	6041633	1849780
Dtcov-32Y-m	H21	Healthy	Pharyngeal	F	26	Healthy	0	T0	No	No	36.216	20624650	2537468
Dtcov-33B-m	H6	Healthy	Faeces	M	32	Healthy	117	T1	No	No	31.075	32552976	2024549
Dtcov-33Y-m	H6	Healthy	Pharyngeal	M	32	Healthy	118	T1	No	No	33.581	1519667	764549
Dtcov-34B-m	H18	Healthy	Faeces	F	23	Healthy	0	T0	No	No	NA	42342325	1629407
Dtcov-34T-m	H18	Healthy	Sputum	F	23	Healthy	0	T0	No	No	31.118	5696996	1067652
Dtcov-35B-m	H17	Healthy	Faeces	F	11	Healthy	0	T0	No	No	NA	42369743	6225507
Dtcov-35Y-m	H17	Healthy	Pharyngeal	F	11	Healthy	0	T0	No	No	32.763	2466612	948269
Dtcov-36B-m	H25	Healthy	Faeces	M	42	Healthy	0	T0	No	No	36.253	37489536	2154149
Dtcov-36Y-m	H25	Healthy	Pharyngeal	M	42	Healthy	0	T0	No	No	35.111	6233556	1638647
Dtcov-37B-m	H21	Healthy	Faeces	F	26	Healthy	22	T1	No	No	37.505	42596397	2525068
Dtcov-37T-m	H21	Healthy	Sputum	F	26	Healthy	20	T1	No	No	25.268	32540137	4904961
Dtcov-37Y-m	H21	Healthy	Pharyngeal	F	26	Healthy	20	T1	No	No	27.426	27745163	4105850
Dtcov-38B-m	H30	Healthy	Faeces	F	75	Healthy	0	T0	No	No	33.245	49568328	2891011
Dtcov-38Y-m	H30	Healthy	Pharyngeal	F	75	Healthy	0	T0	No	No	35.359	1910405	929647
Dtcov-39B-m	H13	Healthy	Faeces	F	4	Healthy	0	T0	No	No	NA	44058820	2177560
Dtcov-39Y-m	H13	Healthy	Pharyngeal	F	4	Healthy	0	T0	No	No	37.153	12698619	2001454
