# Supplementary material for: Assessment of microbiota in the gut and upper respiratory tract associated with SARS-CoV-2 infection
Source: Microbiome. 2023 Mar 3;11:38. doi: 10.1186/s40168-022-01447-0 (PMC9982190; doi:10.1186/s40168-022-01447-0)
Supplement: Supplementary file 4 — Additional file 3: Table S2. Microbes detected in non-template controls. [file 40168_2022_1447_MOESM3_ESM.pdf]

Table S2. Microbes detected in non-template controls

Species	NTC1	NTC2
Achromobacter xylosoxidans	4	8
Acinetobacter calcoaceticus/baumannii complex	8	2
Acinetobacter johnsonii	6	2
Acinetobacter junii	20	12
Actinomyces pacaensis	10	1
Anoxybacillus flavithermus	2	8
Aquabacterium olei	12	5
Burkholderia cepacia complex	2	9
Caldicellulosiruptor acetigenus	11	11
Comamonas thiooxydans	6	3
Cupriavidus gilardii	2	8
Cutibacterium acnes	67	304
Cutibacterium granulosum	1	7
Escherichia coli	8	28
Klebsiella pneumoniae	7	8
Lacimicrobium alkaliphilum	0	10
Micrococcus luteus	3	32
Moraxella osloensis	28	5
Nocardiopsis dassonvillei	0	8
Paracoccus contaminans	0	6
Prevotella melaninogenica	10	2
pseudomallei group	1	6
Pseudomonas balearica	1	13
Pseudomonas fluorescens	7	2
Pseudomonas fluorescens group	9	7
Pseudomonas stutzeri group	2	18
Pseudomonas yamanorum	2	12
Pseudonocardia dioxanivorans	0	6
Rothia mucilaginosa	27	8
Saccharothrix espanaensis	0	6
Schaalia meyeri	10	2
Sinomonas atrocyanea	0	6
Sphingobium yanoikuyae	2	10
Staphylococcus epidermidis	2	13
Stenotrophomonas maltophilia	9	8
Stenotrophomonas maltophilia group	9	8
Streptococcus parasanguinis	8	0
Streptomyces actuosus	0	13
Streptomyces albidoflavus	0	53
Streptomyces albidoflavus group	0	55
Streptomyces alboflavus	0	7
Streptomyces albulus	0	6
Streptomyces albus	0	11
Streptomyces alfalfae	0	9
Streptomyces ambofaciens	0	127

Streptomyces atratus	0	54
Streptomyces aurantiacus group	0	70
Streptomyces avermitilis	0	28
Streptomyces bingchenggensis	0	8
Streptomyces cadmiisoli	0	29
Streptomyces cattleya	0	20
Streptomyces chartreusis	0	22
Streptomyces clavuligerus	0	11
Streptomyces collinus	0	33
Streptomyces dengpaensis	0	24
Streptomyces fodineus	0	17
Streptomyces formicae	0	8
Streptomyces fungicidicus	0	53
Streptomyces gilvosporeus	0	7
Streptomyces glaucescens	0	19
Streptomyces globisporus	0	11
Streptomyces globosus	0	16
Streptomyces griseochromogenes	0	29
Streptomyces griseorubiginosus	0	12
Streptomyces griseus group	0	20
Streptomyces hundungensis	0	12
Streptomyces hygroscopicus	0	77
Streptomyces koyangensis	0	29
Streptomyces lavendulae	0	7
Streptomyces leeuwenhoekii	0	60
Streptomyces lincolnensis	0	15
Streptomyces lividans	0	104
Streptomyces lunaelactis	0	11
Streptomyces lydicus	0	13
Streptomyces malaysiensis	0	6
Streptomyces microflavus	0	6
Streptomyces nigra	0	11
Streptomyces niveus	0	63
Streptomyces olivaceus	0	52056
Streptomyces pactum	0	79
Streptomyces parvulus	0	107
Streptomyces peucetius	0	8
Streptomyces pluripotens	0	18
Streptomyces qaidamensis	0	9
Streptomyces rubrolavendulae	0	17
Streptomyces scabiei	0	20
Streptomyces spongiicola	0	16
Streptomyces venezuelae	0	19
Streptomyces vietnamensis	0	23
Streptomyces violaceoruber	0	6
Streptomyces violaceusniger group	0	88
Streptomyces xiamenensis	0	8

Streptomyces xinghaiensis	0	27
Thermoanaerobacterium thermosaccharolyticum	8	6
Thermus aquaticus	17	29
Thermus brockianus	12	7
Thermus parvatiensis	6	5
Thermus scotoductus	11	10
Thermus thermophilus	21	49
unclassified Acidovorax	17	15
unclassified Acinetobacter	0	8
unclassified Brevundimonas	0	8
unclassified Halomonas	7	13
unclassified Pseudomonas	6	14
unclassified Rhodococcus	0	10
unclassified Sphingobium	6	14
unclassified Streptomyces	0	690
Veillonella parvula	16	2
Xanthomonas campestris	1	13
